# Association of digital measures and self-reported fatigue: a remote observational study in healthy participants and participants with chronic inflammatory rheumatic disease

**DOI:** 10.3389/fdgth.2023.1099456

**Published:** 2023-06-22

**Authors:** Chaitra Rao, Elena Di Lascio, David Demanse, Nell Marshall, Monika Sopala, Valeria De Luca

**Affiliations:** ^1^ Translational Medicine, Novartis Institutes for Biomedical Research, Basel, Switzerland; ^2^ Global Drug Development, Novartis Pharma AG, Basel, Switzerland; ^3^ Research and Insights, Evidation Health, Inc., San Mateo, CA, United States

**Keywords:** fatigue, wearable, digital biomarkers, digital health, multimodal technology, quality of life

## Abstract

**Background:**

Fatigue is a subjective, complex and multi-faceted phenomenon, commonly experienced as tiredness. However, pathological fatigue is a major debilitating symptom associated with overwhelming feelings of physical and mental exhaustion. It is a well-recognized manifestation in chronic inflammatory rheumatic diseases, such as Sjögren’s Syndrome and Systemic Lupus Erythematosus and an important predictor of patient’s health-related quality of life (HRQoL). Patient reported outcome questions are the key instruments to assess fatigue. To date, there is no consensus about reliable quantitative assessments of fatigue.

**Method:**

Observational data for a period of one month were collected from 296 participants in the United States. Data comprised continuous multimodal digital data from Fitbit, including heart rate, physical activity and sleep features, and app-based daily and weekly questions covering various HRQoL factors including pain, mood, general physical activity and fatigue. Descriptive statistics and hierarchical clustering of digital data were used to describe behavioural phenotypes. Gradient boosting classifiers were trained to classify participant-reported weekly fatigue and daily tiredness from multi-sensor and other participant-reported data, and extract a set of key predictive features.

**Results:**

Cluster analysis of Fitbit parameters highlighted multiple digital phenotypes, including sleep-affected, fatigued and healthy phenotypes. Features from participant-reported data and Fitbit data both contributed as key predictive features of weekly physical and mental fatigue and daily tiredness. Participant answers to pain and depressed mood-related daily questions contributed the most as top features for predicting physical and mental fatigue, respectively. To classify daily tiredness, participant answers to questions on pain, mood and ability to perform daily activities contributed the most. Features related to daily resting heart rate and step counts and bouts were overall the most important Fitbit features for the classification models.

**Conclusion:**

These results demonstrate that multimodal digital data can be used to quantitatively and more frequently augment pathological and non-pathological participant-reported fatigue.

## 1. Introduction

Without a doubt, an experience of fatigue, a phenomenon most people are familiar with, is a subjective matter. However, the reasons for fatigue vary (1). Tiredness after a long, physically or mentally demanding day is a sign to rest, so that we can carry on the next day as normal. Such is an experience of most healthy individuals. However, in disease, especially in chronic illnesses like cancer, neurological or autoimmune disorders, fatigue may be exhausting, constantly present and debilitating. It may interfere with ability to function (2–6). This feeling of chronic exhaustion or pathological fatigue is very poorly understood (7).

Symptoms of fatigue in individuals suffering from inflammatory rheumatic diseases, such as Sjögren’s Syndrome (SjS) and Systemic Lupus Erythematosus (SLE) (8) are often major, debilitating, and have significant impact on daily life causing functional limitation and morbidity (9, 10). Fatigue in SLE is found to be associated with increased likelihood of poor sleep quality, depression and anxiety (11, 12). SjS participants also complain of chronic and intractable fatigue, not evident to others (13). Individuals suffering from both SjS and SLE have reported reduced physical activity levels, exercise and aerobic capacities, and muscle weakness, which might be attributed to fatigue (14, 15).

While the impact of fatigue on health-related quality of life (HRQoL) in participants with chronic inflammatory rheumatic diseases is high, there is no consensus regarding quantitative approaches which could be employed. The primary instruments to assess fatigue are self-administered patient reported outcome (PRO) questionnaires. Fatigue PRO assessments can be part of a generic, multi-dimensional questionnaire [e.g., the 36-item short-form health survey (SF-36) (16)], included in disease-specific instruments [e.g., EULAR Sjögren’s Syndrome Patient Reported Index (ESSPRI) (17)], the primary target of the PRO instrument [e.g., the extensively validated 13-item Functional Assessment of Chronic Illness Therapy – Fatigue Scale (FACIT-Fatigue) (18)] or Multidimensional Fatigue Inventory (MFI) (19). Fatigue PROs are commonly used in both clinical practice and clinical trials. These instruments can also cover various domains of HRQoL factors (including usual daily activities, mood, sleep) and hence reflect the broad impact of a disease on various aspects of participants’ lives and their overall well-being. Yet PRO data collection has some limitations, which include bias (completion in clinical setting of a hospital or doctor’s office; recall of 1 week or longer; responses influenced by fatigue level at a particular day or moment, inconsistent compliance (when completed at home), and lack of proximity to the disease or treatment (20–22).

In recent years, global availability of affordable digital health technologies, in particular wearable devices, has gained tremendous interest in the clinical field, offering a unique opportunity to collect more frequent, continuous, and objective data on activity and patient status (23, 24), which can augment PRO data. Common digital measures focus on physical activity (e.g., number or steps from accelerometers), and vital signs (e.g., heart rate from photoplethysmography). Successful applications of digital measures also include cognition (25, 26), gait and mobility (27), pain (28, 29) and stress (29). A feasibility study was previously conducted on healthy participants to explore the relationship between self-reported, non-pathological physical and mental fatigue and behavioural and physiological time-series data acquired from a multisensor wearable device (30). Results from this study suggested that data from multiple sensors are needed simultaneously to classify and characterize subjective, non-pathological fatigue.

In this study, we have applied a similar approach on a larger observational dataset, which included data from healthy individuals, and individuals diagnosed with chronic inflammatory rheumatic diseases: SLE and SjS. Descriptive statistics, supervised and unsupervised machine learning approaches were used to characterize patient groups and the association between daily behaviors (using passively-collected wearable device data) and self-reported fatigue. The key contributions of this work are: (i) the characterization and exploration of differences in physical activity and reported HRQoL factors, including fatigue, in healthy volunteers (HV) vs. participants with chronic inflammatory rheumatic diseases by using both digital sensor measures and participant-reported data; (ii) development of a machine learning-based framework to combine multi-sensor and participant reported outcome data to classify participant-reported scores on fatigue and hence (iii) identify a set of key predictive metrics of fatigue. These multi-modal, composite metrics may be further explored in the context of clinical trials as digital biomarkers or patient stratification criteria.

## 2. Materials and methods

### 2.1. Data

Observational data were acquired by Evidation Health, Inc.^1^ between August and October 2020 from a total of 296 individuals (19–80 years of age, mean age 45) in the United States, of whom 105 were HV, 104 SLE and 87 SjS participants. Each individual participated in the study for a period of one month. Survey data on demographics, medical history, symptoms and current treatment for autoimmune disease(s) have been collected at baseline. All participants were English speakers. Financial incentives of up to $47 were provided for participation to the study. The compensation for completing the baseline survey and connecting the Fitbit device to the Evidation Achievement platform was of $10. The study was approved by the relevant Institutional Review Boards and written consent was obtained from every participant. An overview of the study population is provided in Table 1 and Figures S1–S4 in Supplementary Material A. A higher percentage of female participants were recruited in this study (overall percentage of female in the data set is 90.9%) to reflect the fact that both SLE and SjS are overrepresented diseases in women, with the sex ratio of approximately 9:1 female to male (31, 32).

**Table 1:** Study cohorts characteristics including age, sex and ethnic background.

	HV	SLE	SjS	Total	Overall percentage
Number of participants	105	104	87	296	–
Sex					
Female	79	103	87	269	90.9
Male	26	1	0	27	9.1
Mean age ± sd	43.9±11.1	43.2±11.2	43.9±9.4	45.4±11.0	–
Race					
American Indian or Alaska Native	2	4	0	6	2.0
Asian	7	6	2	15	5.1
Black	14	10	8	32	10.8
Native Hawaiian Pacific Islander	1	0	0	0	0.3
White	84	87	76	247	83.4
Other	4	3	1	8	2.7
Ethnicity					
Non-Hispanic Latino	94	92	83	269	90.9
Hispanic Latino	11	12	4	27	9.1

HV: healthy volunteers; SLE: systemic lupus erythematosus; SjS: Sjögren’s syndrome; sd: standard deviation.

Objective, continuous data on physical activity, sleep and heart rate (HR) were collected over one month using participants’ owned multi-sensor wearable devices from Fitbit.^2^ Minute-by-minute Fitbit data were divided into three categories (HR, sleep and steps) and multiple features were provided or computed at different time resolutions (monthly, weekly, daily and hourly). In this work, we focused on 93 daily-aggregated Fitbit features. All Fitbit features are listed in the Supplementary Material B, Tables S3–S5.

In addition to providing their wearable Fitbit device data, participants were asked to answer to daily and weekly lists of questions, see Sections 3.1 and 3.2 in Supplementary Material A. The questions aimed to collect the participants perspective and own assessment on various HRQoL factors and symptoms, namely pain, mood, general physical activity and functioning, sleep, physical (PhF) and mental fatigue (MF), and daily tiredness (T). Questions were administered via mobile app. Daily questions were assigned to the same calendar day as the daily Fitbit data. The daily list consisted of a total of 9 questions with some reporting both intensity and frequency of the HRQoL factor. Weekly list of 17 questions included 13 question of the validated FACIT-Fatigue scale v4 (under license from FACIT.org^3^) (18). The remaining 4 questions were related to mood, physical (PhF) and mental fatigue (MF). The last two were as follows:

∙ Physical fatigue score (PhF): Physically, in the last week how often did you feel exhausted?
  Possible answers: “never”, “sometimes”, “regularly”, “often”, “always”
∙ Mental fatigue score (MF): Mentally, in the last week how often did you feel exhausted?
  Possible answers: “never”, “sometimes”, “regularly”, “often”, “always”

Except for FACIT-Fatigue, this survey did not rely on validated clinical outcome measures. We will therefore refer to the categorical answers to each question of the daily and weekly list of questions as participant-reported (PR) features.

### 2.2. Data pre-processing

To ensure high quality data for our analysis, we excluded data from participants with low adherence. Only 207 of the involved participants worn the Fitbit device during the study. The total number of days available for the Fitbit users before data cleaning was 6,992. Regarding participant-reported data, 293 participants filled in at least one daily list of questions and 272 participants have at least 15 overall days of completed list of questions. Additional information about study adherence is reported in Section 2 of Supplementary Material A. For each participant and monitored day, we included only days when (i) the wearable device was worn for at least 60% (empirical threshold) of the total wear-time computed as the number of minutes in which the fitbit was worn and recording data; (ii) participants responded to the complete daily list of questions and (iii) the corresponding weekly one. Furthermore, missing values in any feature categories were excluded from the analysis. This step resulted in an exclusion of 113 participants. Data from a total of 183 participants (68 HV, 57 SjS and 58 SLE) were used for further analysis.

93 digital features (33 HR, 27 sleep, 33 steps) were computed by aggregating fitbit data at the day level. PR features, corresponding to the daily questions without daily tiredness, were derived. This set of features was used for the classification tasks of weekly fatigue and daily tiredness. Dimensionality reduction was performed to extract features carrying higher variance and hence to lower the size of the data while retaining interpretability. For this purpose, we used principal component analysis (PCA) on the numerical daily Fitbit features for each set (HR, sleep and steps) independently. The numerical features were normalized to have standard deviation equal to 1. A threshold of 80% for the cumulative percentage of total variance was set to select the top principal components (PCs). Factor analysis of mixed data (FAMD) was applied to the categorical daily PR features. Similarly to PCA, we used the threshold of 80% for the cumulative percentage of total variance to select the top PCs. Further method details are reported in Section 5 of the Supplementary Material B. The dimension of the feature space was finally reduced from a total of 93 to 65 digital features (23 HR, 21 sleep, 21 steps) and 6 daily PR features. The features contributing to most of the variances in the top PCs were then considered for descriptive statistics and clustering of digital features. The full list of Fitbit features and PR features is provided in the Supplementary Material B (Tables S2–S5).

### 2.3. Descriptive statistics and clustering of digital features

For each of the 65 PCA-selected daily digital features, we computed participant-specific descriptive statistics over the observed period of time. In our analysis, we considered a single day for each participant as an independent observation. Statistics were conducted by comparing the observations (days) computed for different participant groups based on diagnosis (HV, SjS and SLE). Data was checked for normality using Kolmogorov–Smirnov test with p-value <0.05, indicating that the majority of Fitbit features in each participant group were not normally distributed. Non-parametric test Kruskal–Wallis one-way ANOVA test was then used to highlight significant differences of each feature among the three participant groups. Post hoc pairwise analysis was performed with the non-parametric Dunn’s test to identify differences between two groups, p-values were adjusted using the Holm–Bonferroni method. Finally, the association between daily PR features, including tiredness, was measured with Goodman and Kruskal’s τ test.

To further investigate the relationship between objective behavioral data and subjective fatigue scores and extract potential digital phenotypes, Ward’s agglomerative hierarchical clustering with Euclidean distance was applied on the 65 daily Fitbit features for 3950 observed days, similarly to (30). The number of clusters and feature sets were determined based on the hierarchical structure of the dendrograms showed in Figures S32 and S33 in Supplementary Material C.

### 2.4. Classification of participant-reported fatigue

Two classification tasks were carried out to demonstrate whether subjective fatigue scores reflecting both pathological and/or non-pathological fatigue can be linked to digital parameters from wearable devices and other survey data. Extreme Gradient Boosting (XGBoost) (33) classifiers were trained to classify (i) binary labels of weekly physical (PhF) and mental fatigue (MF), independently; and (ii) multi-class labels of daily tiredness score (T).

For binary classification, the different levels of MF and PhF fatigue questions were converted into binary labels $y_{MF}$ and $y_{PhF}$, respectively, using the following criteria:

$$y=\left\{ \begin{array}{cc} 0\; & \;\mathrm{for}\;\mathrm{MF}\;\mathrm{or}\;\mathrm{PhF}=\text{never}\;\\ 1\; & \;\mathrm{for}\;\mathrm{MF}\;\mathrm{or}\;\mathrm{PhF}\in \{\mathrm{sometimes};\;\mathrm{regularly};\;\mathrm{often};\;\mathrm{always}\} \end{array}\right.$$

The weekly questions were replicated retrospectively over the 7 days prior the day of the questions to match the observation period of the respective question. The day of the questions was included only if the questions had been completed after 6 p.m. The predictions for weekly PhF and MF were made for each day.

For multi-class classification, the different levels of daily tiredness (T) were encoded as ordinal to $y_{T}$:

$$y_{T}=\left\{ \begin{array}{cc} 1\; & \;\mathrm{for}\;T=\mathrm{not}\;\mathrm{at}\;\mathrm{all}\;\\ 2\; & \;\mathrm{for}\;T=a\;\mathrm{little}\;\mathrm{bit}\;\\ 3\; & \;\mathrm{for}\;T=\mathrm{some}\;\mathrm{what}\;\\ 4\; & \;\mathrm{for}\;T=\mathrm{quite}\;a\;\mathrm{bit}\;\\ 5\; & \;\mathrm{for}\;T=\mathrm{very}\;\mathrm{much}\;\; \end{array}\right.$$

Input features for both classification tasks were: (i) the 8 PR features, corresponding to the daily questions excluding reported daily tiredness; (ii) 93 Fitbit features; and (iii) participant diagnosis (HV, SjS or SLE) and age. Different combinations of features sets were tested to determine the effect of the modalities on the classification performance. To evaluate the models a *leave one group out* (LOGO) validation strategy was used. In LOGO, observations (i.e., single days) in the train and test sets are stratified by label distribution, grouped by participants, and repeated five times guaranteeing that data of the same participants are not in the two sets concurrently. Similar proportion of observations across HV, SjS and SLE groups was maintained in the train and test sets. The LOGO strategy is participant-independent and enables to test the ability of the model to generalize to unseen participants. Within the train set we used a grid-search stratified 5-fold cross-validation (CV) scheme to derive the set of hyperparameters of the XGBoost that maximises the balanced accuracy. Due to the skewed distributions of labels, balanced accuracy and F1-score metrics were used to evaluate the performance of the weekly fatigue classification. Balanced accuracy, unweighted average of the per-class F1-score and the macro-averaged mean absolute error (MMAE) were used to evaluate the performance of the daily tiredness classification. Further details on experimental setup, hyperparameter tuning, validation, model evaluation and implementation are described in the Supplementary Material C, Section 8.

## 3. Results

### 3.1. Characterization of HV vs. participants with chronic inflammatory rheumatic diseases using statistical analysis

This section reports results from the statistical analysis described in Section 2.3 for the characterization of HV vs. SjS and SLE patients. On average, daily PR features indicated overall poorer scoring in all HRQoL factors in disease (SjS and SLE) cohorts compared to healthy participants. An overview of all daily answers to the daily survey, stratified by participant groups is provided in Figure S8 in Supplementary Material A. Low association was found between survey domains (τ<0.3), yet association was high between the same domain’s intensity and frequency (τ>0.6). The full association map can be found in the Supplementary Material A, Figure S9.

**Figure 1:**
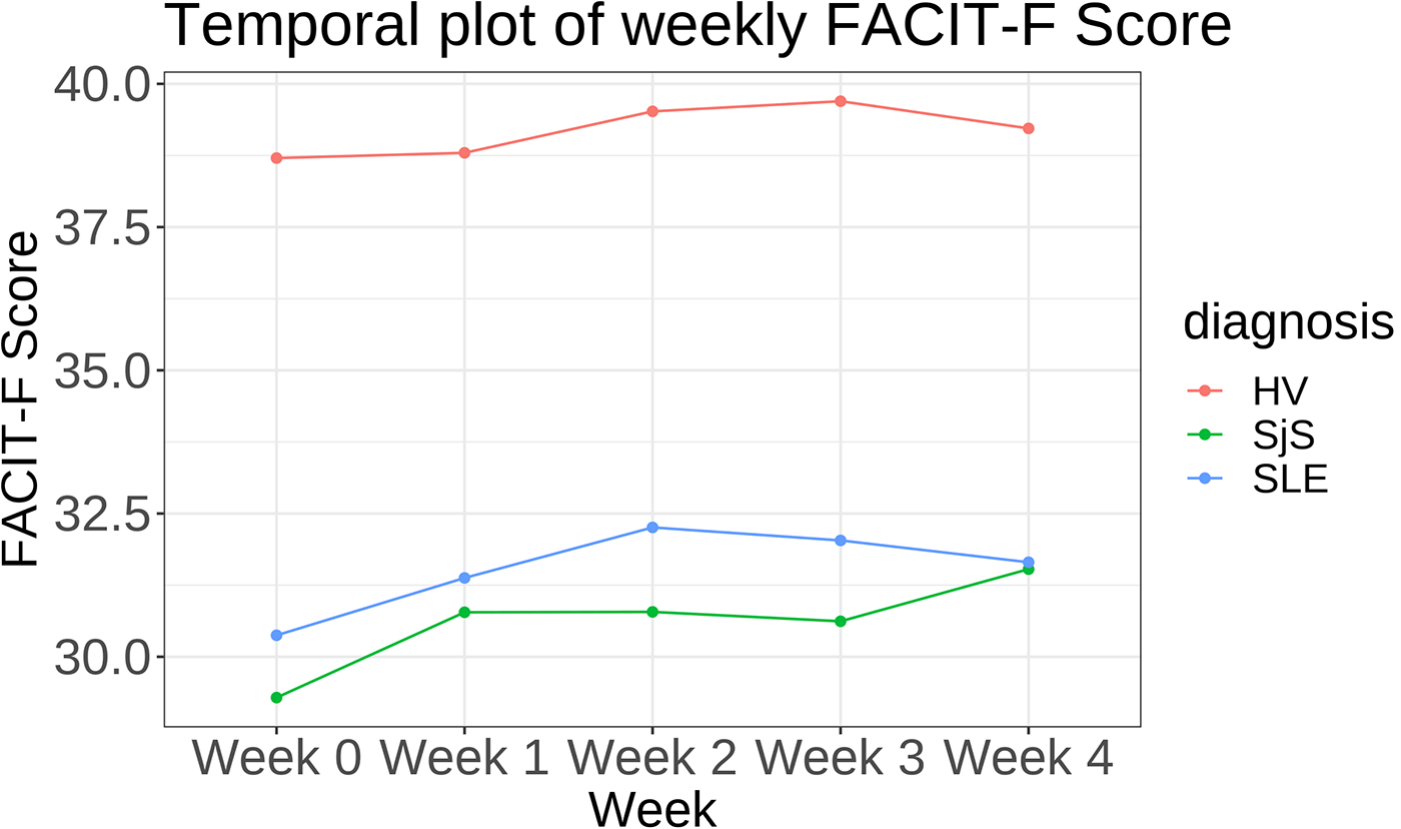
Temporal plot showing mean FACIT-Fatigue (FACIT-F) score per week stratified by disease cohort.

Participants with SjS and SLE reported on average lower FACIT-Fatigue scores and hence higher levels of fatigue^4^ compared to healthy participants. SjS participants reported the lowest FACIT-Fatigue score (mean score of 30.6±0.8), followed by SLE (31.5±0.7), and HV (39.2±0.4). Difference in fatigue scores was consistent between participant groups among all time points (see Figure 1). Similar trends but not substantially different between SjS and SLE participants were observed for weekly MF and PhF scores and daily tiredness scores. Figure S13 in Supplementary Material A shows the ratio of different levels of PhF and MF across participant groups and Supplementary Material A, Figure S8 (bottom right) the different levels of daily tiredness across cohorts.

**Figure 2:**
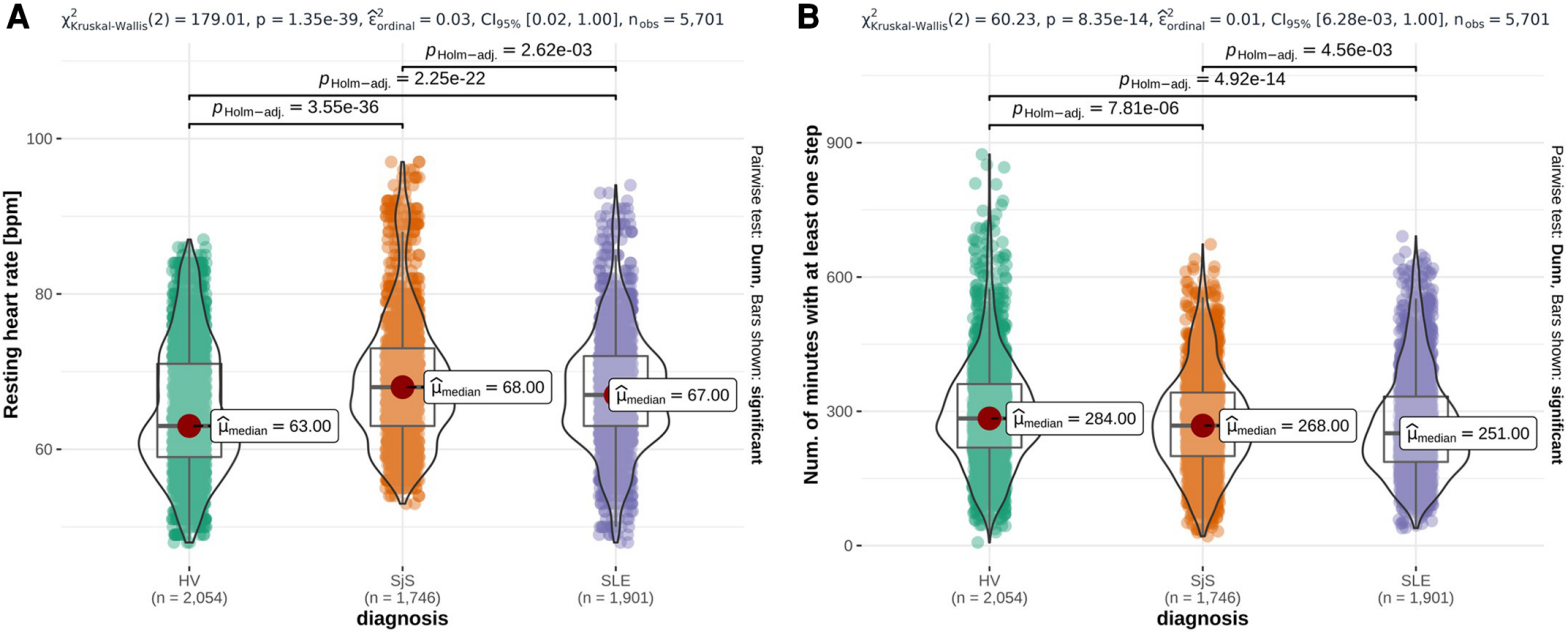
Overlay of violin plots and box plots for three representative Fitbit features that are significantly different among participant groups (HV, SjS and SLE), namely daily mean resting heart rate (**A**), number of minutes with at least one step (**B**). p-values from Dunn’s pairwise test, adjusted using the Holm–Bonferroni method, are reported on the top of each plot and the number of observations (n) at the bottom. Red dots highlight the participant group median values (${\hat{\mu }}_{median}$).

From the PCA-filtered Fitbit features, eight out of 23 HR features and two out of 21 steps features were statistically significantly different (p-value < 0.001) across all three studied groups, while no sleep features were significantly different across all three studied groups. Figure 2 shows two examples, namely resting HR, which was the lowest in HV and highest in SjS participants, and the number of minutes with at least one step, which was the lowest in SLE and SjS participants and highest in HVs. Figures S21–S31 in Supplementary Material B show the overlay of violin and box plots for all 65 PCA-filtered Fitbit features.

### 3.2. Description of digital phenotypes using clustering of wearable features

**Figure 3:**
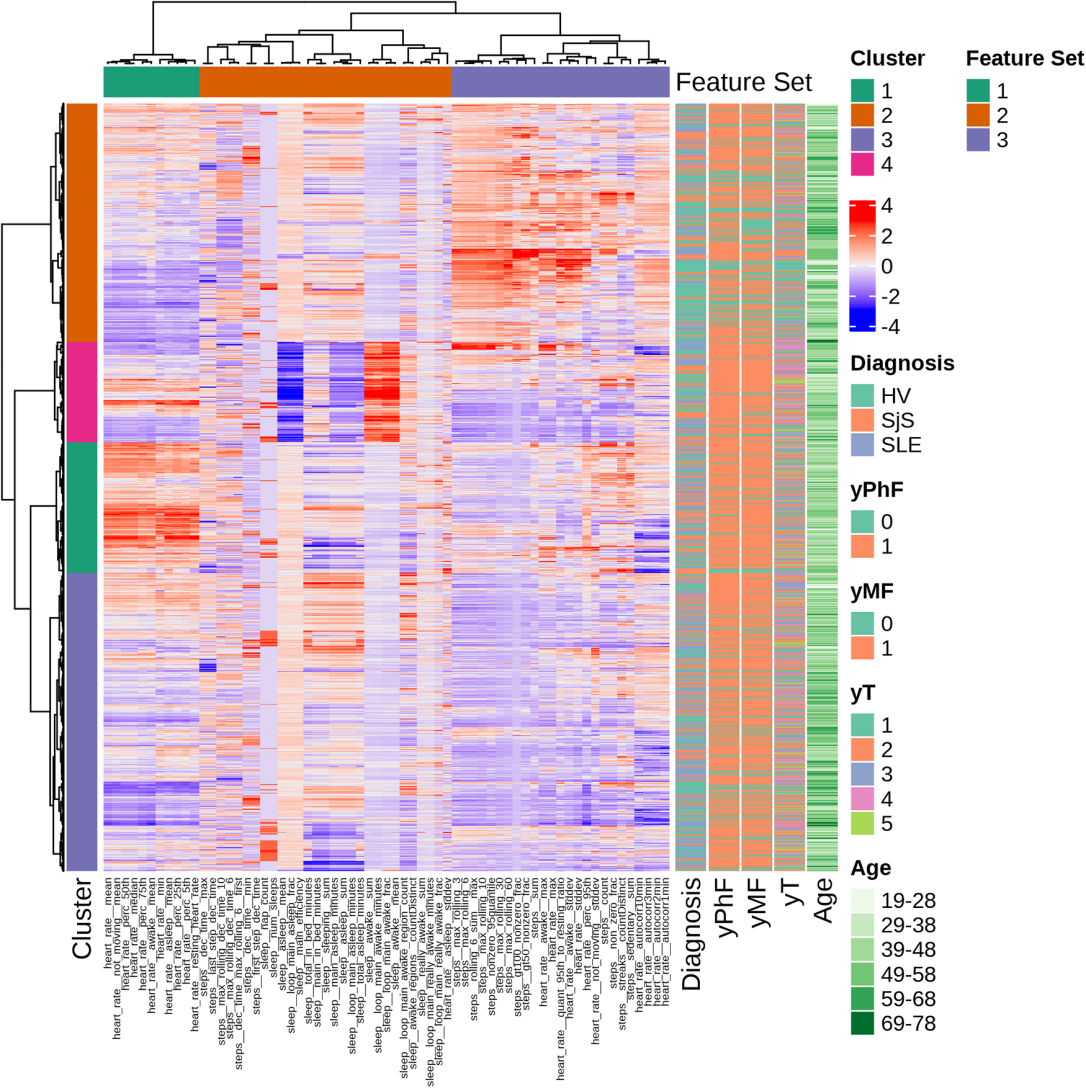
Hierarchical clustering overview with dendrograms and heatmap of ranked features. Each row corresponds to one observation and each column to the Z-score normalized values of the 65 Fitbit features. Corresponding PhF, MF and T labels, age and diagnosis are displayed in the annotations on the right.

This section reports results from the clustering analysis (see Section 2.3) for the description of the identified digital phenotypes. Figure 3 shows the output of hierarchical clustering of the daily wearable features, where each row corresponds to one observation (monitored day per participant) and each column the Z-score normalized values of the 65 Fitbit features. For each observation, the corresponding physical ($y_{PhF}$) and mental fatigue ($y_{MF}$), and daily tiredness ($y_{T}$) labels, and demographics are reported. We observed 3 groups of features and 4 clusters of 517, 671, 1227 and 1535 observations each.

Feature set 1 consists only on HR features (e.g., mean resting and daily HR). Sleep features are all clustered in feature set 2, along with a HR feature (i.e., HR standard deviation while asleep) and steps features. Feature set 3 has a combination of steps-based and heart rate features, mostly related to physically active periods (e.g., walking bouts and maximum number of steps in specified time windows).

Four clusters have been identified. Cluster 1 is mainly composed of observations from SLE (18% of total SLE observations) and SjS (21% of total SjS observations) and of participants belonging to middle age groups (29-58 years old). This cluster reports low physical activity, higher HR compared to other clusters and high levels of daily tiredness. Cluster 2 groups observations characterized by high values for physical activity parameters. This cluster includes more HVs (39% of total HV observations) compared to disease groups (SjS and SLE) and 48% of observations with no reported fatigue are in this cluster. Daily tiredness levels in this cluster are comparatively low. Cluster 3 is driven by very low values for HR and physical activity parameters and includes a similar proportion of HV, SLE and SjS participants. Furthermore, it has the highest proportion (73%) of participants belonging to the oldest age group, that is 69-78 years of age. Finally, cluster 4 is characterized by high values for sleep parameters during awake (daytime) periods and low scores for sleep efficiency.

Statistically significant differences of selected Fitbit features and distribution of survey answers for each domain in the four clustered groups are displayed in Figures S36 and S37 in Supplementary Material C, respectively. Within-participant observations were split across the different clusters and details are provided in Figure S38 in Supplementary Material C.

### 3.3. Machine learning-based framework to classify participant-reported weekly fatigue and daily tiredness

In this section, we report the main results from the classification analysis described in Section 2.4 in terms of classification performance and feature importance, the latter to highlight the key predictive metrics of of participant-reported fatigue.

Tables 2 and 3 summarize respectively the binary classification results on the test set for predicting binary labels of weekly PhF and MF. In both classification tasks the highest performance was achieved when using multimodal data from Fitbit, PR features and diagnosis state, with a balanced accuracy of 0.78±0.08 and 0.76±0.05 for PhF and MF respectively. The F1-score of the positive class ($F1_{1}$), achieved by the best model, was of 0.87±0.03 and 0.84±0.03 for PhF and MF respectively.

**Table 2:** Classification results of binary labels of weekly physical fatigue $y_{PhF}$.

Input features	Balanced accuracy	$F1_{0}$	$F1_{1}$
PR	0.76±0.07	0.50±0.09	0.79±0.08
HR	0.55±0.03	0.28±0.04	0.73±0.06
Sleep	0.54±0.02	0.27±0.02	0.71±0.05
Steps	0.57±0.04	0.29±0.05	0.78±0.03
All Fitbit	0.61±0.06	0.35±0.07	0.77±0.05
All Fitbit + PR	0.78±0.04	0.56±0.08	0.86±0.06
All Fitbit + Demographics	0.63±0.12	0.37±0.16	0.83±0.05
All Fitbit + PR + Demographics	0.78±0.08	0.55±0.10	0.87±0.03

Evaluation metrics are summarized as the cross-validation mean ± standard deviation. The top two set of results are highlighted in bold font.

F1: F1-score; PR: features from participant-reported data (daily questions); HR: heart rate features (Fitbit).

**Table 3:** Classification results of binary labels of weekly mental fatigue $y_{MF}$.

Input features	Balanced accuracy	$F1_{0}$	$F1_{1}$
PR	0.73±0.04	0.52±0.04	0.77±0.03
HR	0.54±0.02	0.32±0.03	0.7±0.07
Sleep	0.52±0.01	0.28±0.03	0.72±0.06
Steps	0.59±0.03	0.37±0.03	0.77±0.04
All Fitbit	0.58±0.04	0.36±0.05	0.73±0.07
All Fitbit + PR	0.74±0.05	0.54±0.05	0.82±0.02
All Fitbit + Demographics	0.59±0.08	0.34±0.12	0.77±0.05
All Fitbit + PR + Demographics	0.76±0.05	0.57±0.06	0.84±0.03

Evaluation metrics are summarized as the cross-validation mean ± standard deviation. The top two set of results are highlighted in bold font.

F1: F1-score; PR: features from participant-reported data (daily questions); HR: heart rate features (Fitbit).

**Figure 4:**
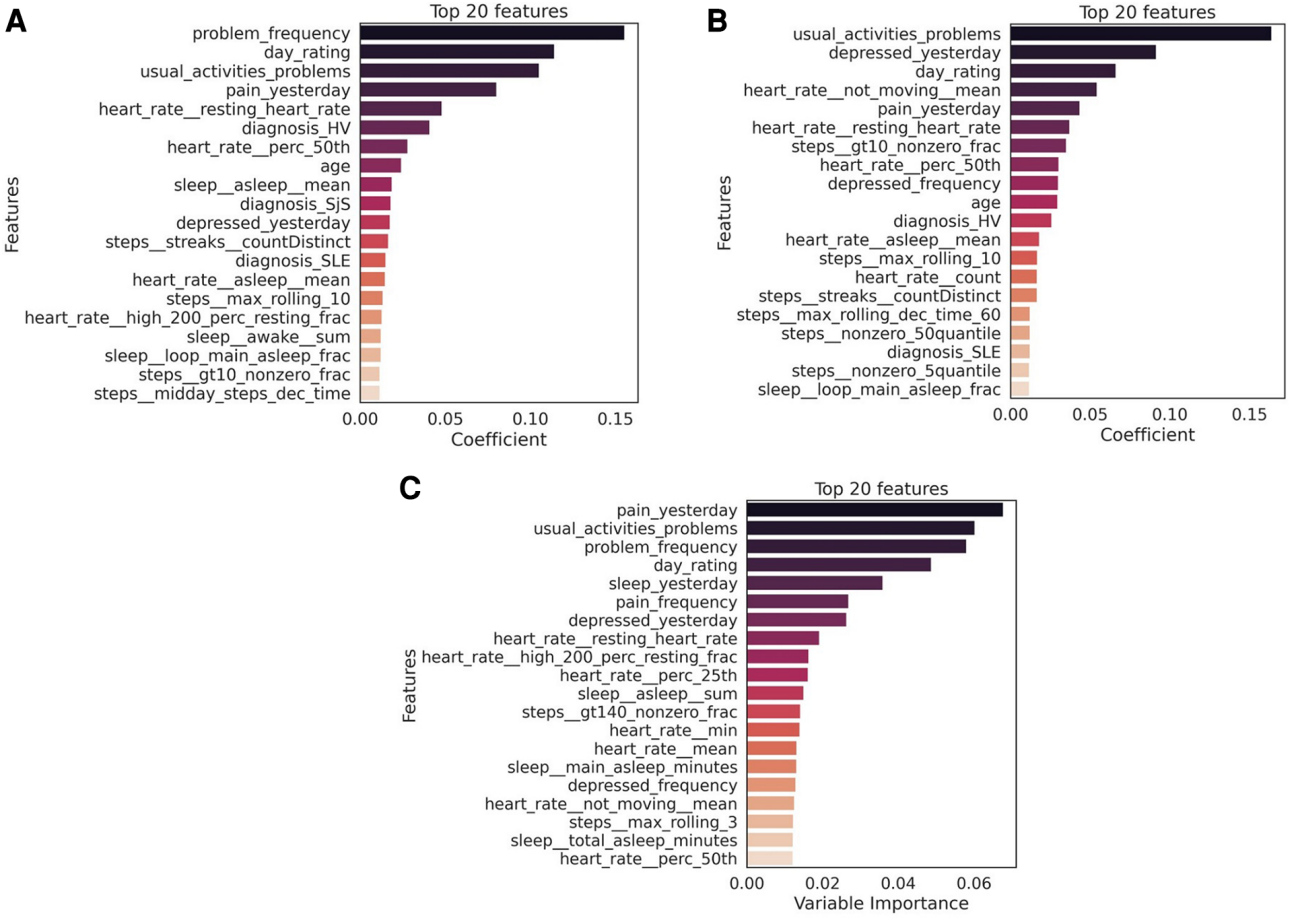
Top 20 features to classify (**A**) physical fatigue (PhF), (**B**) mental fatigue (MF) and (**C**) daily tiredness (T). The feature importance was extracted from the best performing model for each task (PhF and MF trained on all Fitbit, PR and diagnosis features; daily tiredness: trained on all Fitbit and PR features). The mean importance of each feature across the LOGO iterations, is measured as the mean of the average gain across the trees splits in which the features was used. The higher the value of the gain score, the higher importance of that feature in the model. Bars in the graph are colored based on the importance coefficient, the higher the importance the darker the color.

Using daily PR features alone led to a decrease in performance especially of $F1_{1}$ (about 7% for both MF and PhF), while using Fitbit features alone substantially decreased the balanced accuracy for both tasks (17% decrease for PhF and 83% for MF). Feature importance from the XGBoost classifier was extracted for all experiments, see Figures 4A,B for predicting PhF and MF, respectively. For the best performing XGBoost classifier for PhF classification (trained on all features), the top 5 important features include pain PRs, PR features related to problems in performing daily activities and resting HR. For MF, the top 5 important features of the best XGBoost classifier included PR features related to depressed-like mood and reporting problems in performing daily activities, followed by HR during rest or sedentary behaviors. In the overlapping top 20 important features to classify both PhF and MF labels using the best performing models, there were 6 PR, 6 HR and 6 steps and 2 sleep features, see Figure S40 in Supplementary Material C. Some of the features were specific to predicting only PhF (e.g., the diagnosis state of SjS, proportion of minutes in asleep state) and others specific to MF (e.g., depressed-like mood PR, mean heart rate while not moving).

**Figure 5:**
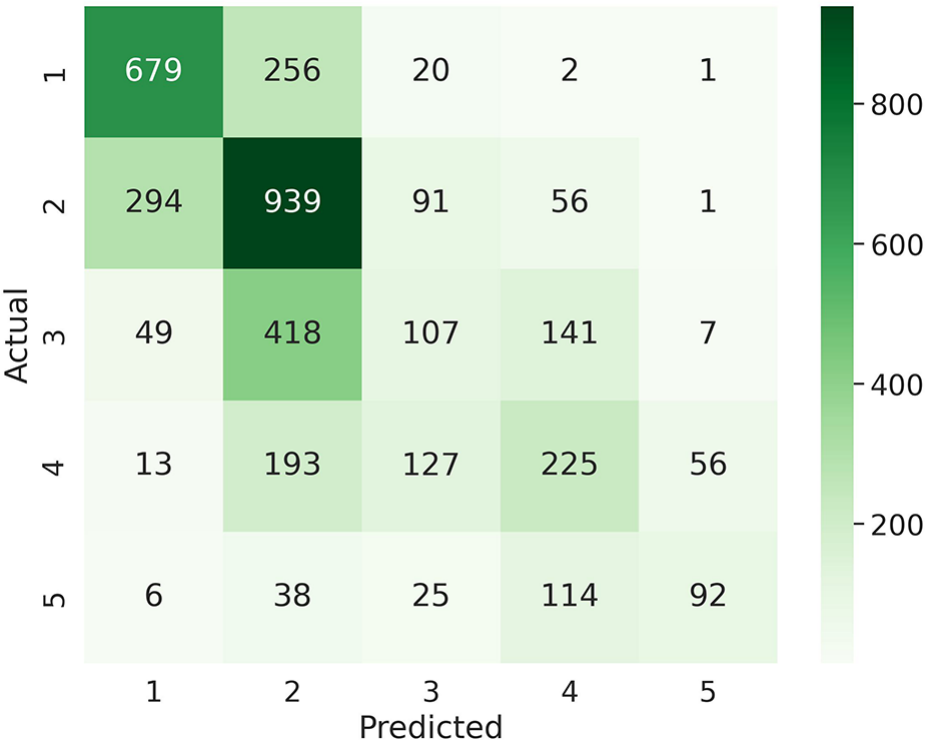
Confusion matrix for multi class classification of daily fatigue labels.

Table 4 summarizes the multiclass LOGO classification results for predicting labels of daily tiredness $y_{T}$. Similar experiments as for binary classification were performed by subsetting input features. Similar performance was achieved when using PR features alone or in combination with all Fitbit. Specifically, PR features only achieved a balanced accuracy of 0.46±0.03, a mean F1 score of 0.47±0.02 and a macro-averaged MAE of 0.72±0.04, when combined with Fitbit features a balanced accuracy of 0.46±0.02, a mean F1 score of 0.46±0.03 and an MMAE of 0.74±0.05. The confusion matrix of the experiment combining PR and Fitbit features is shown in Figure 5. Among extreme errors, six observations belonging originally to class $y_{T}=5$ (T = “very much” tired) were falsely classified as class 1 (T = “not tired at all”). These observations belong to 3 HV and 2 SLE participants. PR features for these observations showed low scores, indicating overall good HRQoL. Distributions of top 10 features of the 1 and 5 classes as well as misclassified observations are shown in Figure S41 in Supplementary Material C. Although not strongly associated to PR daily tiredness scores (see Figure S9 in Supplementary Material A), both activity performance and pain PR scores showed higher importance to classify daily tiredness.

**Table 4:** Classification results of multi class labels of daily fatigue $y_{T}$.

Input features	Balanced accuracy	F1	MMAE
PR	0.46±0.03	0.47±0.02	0.72±0.04
HR	0.21±0.01	0.18±0.03	1.47±0.04
Sleep	0.23±0.01	0.21±0.01	1.39±0.08
Steps	0.24±0.02	0.23±0.03	1.38±0.05
All Fitbit	0.23±0.02	0.21±0.02	1.38±0.07
All Fitbit + PR	0.46±0.02	0.46±0.03	0.74±0.05
All Fitbit + Demographics	0.24±0.03	0.23±0.03	1.34±0.08
All Fitbit + PR + Demographics	0.45±0.03	0.45±0.03	0.74±0.06

Evaluation metrics are summarized as the cross-validation mean ± standard deviation. F1 score is the unweighted mean of all the classes’ F1 scores. The MMAE is the macro-averaged mean absolute error. The top two set of results are highlighted in bold font.

PR: features from patient-reported data (daily questions); HR: heart rate features (Fitbit).

Mainly HR features (e.g., related resting HR) ranked among the top 20 important Fitbit predictors of daily tiredness, which are listed in Figure 4C.

## 4. Discussion

### 4.1. Characterization of HV vs. participants with chronic inflammatory rheumatic diseases

Results from the statistical analysis of both sensor features and daily questions on the characterization of HV vs. SjS and SLE patients (Section 3.1) highlighted some key differences between groups of participants included in this survey. From Fitbit data, we observed that several features were significantly different among HV, SLE and SjS groups: median resting HR was higher for SjS (68 bpm) and SLE (67 bpm) participants compared to HV group (63 bpm). SLE and SjS groups also showed lower levels of physical activity compared to the HV group. Although relying only on non-clinically validated patient-reported and Fitbit data, this observation is in line with previous studies which suggest that an increased HR (without known cardiovascular diseases) and physical inactivity are associated with systemic inflammation (35, 36). Chronic systemic inflammation is one of the key features of autoimmune rheumatic diseases like SLE and SjS (37, 38). These observations suggest a clear direction of change for these features when quantifying improvement due to treatment, e.g., in clinical trials. Literature confirms sleep is affected in both SjS and SLE. SjS patients report range of sleep disturbances including prolonged sleep onset time and frequent night awakenings (39). Sleep can affect the disease activity in SLE and pain and fatigue in these patients are known to be associated with sleep disorders (40). However, sleep features show small or no substantial difference among disease cohorts in our data.

As expected, SjS and SLE participant groups reported on average higher levels of both physical and mental fatigue and daily tiredness than the HV group, indicating that fatigue and tiredness should be closely monitored and included already in early-phase clinical trials with repeated measures. While there is no validated instruments to assess fatigue in the diseases included in this study, there was a consistency in FACIT-Fatigue scores and self-reported physical and mental fatigue from the weekly questions, as seen by the negative correlation of FACIT-Fatigue score with PhF and MF as well as with daily questions in Supplementary Material A, Figures S13 and S14. Future work could focus on further exploration and collection of data from different instruments to assess fatigue symptoms and derive more sophisticated fatigue labels.

Other HRQoL factors, such as those related to pain and mood, also show on average poor ranking in disease groups (SLE and SjS) compared to HVs. An important point to note here is that the data were collected during the COVID-19 pandemic. Participants did not test positive for COVID during the study, however the pandemic restrictions might have had an impact on their behaviour, further affecting the quality of life in these participants. Eventual changes in behaviors might also limit the generalization of results and wearable device data. No significant association between treatment or medication and participant-reported fatigue levels was found as shown in Figure S14 in Supplementary Material A. In both treated and untreated groups of SLE and SjS participants, the majority reported physical and mental fatigue as “regular”, “often” or “sometimes” present. Five and 11 out 87 participants with SjS had no experience of physical or mental fatigue, respectively, regardless of treatment/no treatment. Always present mental fatigue was experienced by 4 out of 104 participants within SLE and 6 out of 87 participants in SjS group. No significant association was also found between FACIT-Fatigue score and medication, tested using Mann–Whitney U-test, as shown in Figure S15 in Supplementary Material A.

To analyse the variability of reported fatigue in a non-interventional setting, a longitudinal FACIT-Fatigue score analysis was performed using a linear mixed-effect model repeated measures, with diagnosis, sex and analysis visit (week) as factors and baseline, age and FACIT-Fatigue score as continuous covariates. A compound symmetry structure was assumed for this model in order to take into account the within-participant correlation of different measurements. No statistical effects of time (week) from this model was observed. For the daily PR questions a multiple correspondence analysis was performed with all timepoints to assess the association between all items of the daily list of questions. Similarly to FACIT-Fatigue yet less strongly, the results obtained from the this analysis suggested that the first axis carries the main real information. As confirmation, the first axis was strongly associated with the FACIT-Fatigue score. The intra-participant variability of Fitbit measures was higher, possibly due to contextual events affecting daily behaviours. In this work, we computed statistical analyses considering single days for each participant as independent observations. Additional time-aware analyses (e.g., repeated measures ANOVA) and investigation of individual participant trajectories could be further explored in future work.

### 4.2. Digital phenotyping

Results from the cluster analysis of daily Fitbit data on the description of digital phenotypes, reported in Section 3.2 did not reveal disease-specific groups of observations, suggesting that such behavioral data might not be prognostic. Yet we observed different digital, behavioral phenotypes. Cluster 2 is presumably the ’healthy’ phenotype in this dataset with the highest levels of measured physical activity, percentage of HVs and high percentage of observations of no fatigue or tiredness. Cluster 4 can be characterized as ’sleep affected’ phenotype cluster as it includes high values for parameters associated with proportion of sleep minutes in “restless” or “awake” sleep patterns and low scores for sleep efficiency parameters. Previous studies on association of sleep and inflammatory diseases have shown that poor sleep is associated with biomarkers for high level inflammation (41). Interestingly, this cluster also includes more observations with reported fatigue compared to no fatigue, indicating this kind of fatigue might be associated with less sleep or poor sleep efficiency (40). Cluster 1 with participants mostly from middle-age groups can be described as ’fatigue’ phenotype as it includes several days where both PhF and MF and high level of daily tiredness were reported. Fatigue in this cluster is correlated with low physical activity (36) yet higher HR values across all age ranges. Previous studies suggest that increased heart rate is observed in patients suffering from inflammatory diseases (42). Aging is also an important factor in low grade inflammations resulting in reduced physical activity (43).

Distinguishing participants through cluster analysis was not clear. We observed that observations belonging to same participants were often split across the 4 clusters, highlighting the longitudinal variability of participant behaviors and hence phenotype. This is in line with observations of other research groups (44, 45) and may reflect diurnal fluctuations in fatigue, possibly associated with temporal changes as a consequence of sleep, rest, and physical activity throughout the day (46). For future work, aggregating the observations from participants might help us better characterize individual participants or patients and not just behaviours, however a large population is needed for this purpose. Digital phenotyping strategies could be applied to a broader spectrum of complex diseases that require the interpretation of highly multidimensional integrative data through disease and patient stratification. The advantage of longitudinal data collection will be crucial in clinical trials when investigating disease symptoms, and their potential change in response to treatment and after stopping of the treatment.

### 4.3. Classification of weekly fatigue and daily tiredness and identified key predictive metrics

Results reported in Section 3.3 on the classification of weekly fatigue and daily tiredness, show that the highest performance was achieved by XGBoost for both physical and mental fatigue classification when using features from all data modalities. The resulting best set of XGBoost hyperparameters for these configurations are learning rate of 0.05, a maximum depth of 2 and number of threes of 100. By extracting the top predictive features from the best performing model to classify weekly PhF and MF, we observed that from digital data, mostly physical activity (e.g., maximum rolling of step) and HR (e.g., resting HR) contributed to the classification while only three sleep features (e.g., proportion of asleep minutes, minutes of “restless” and “awake” sleep patterns and fraction of minutes during the main sleep spent in “asleep” sleep pattern) were present. This suggests that multimodal data are necessary towards a quantitative assessment of fatigue. The top predictive features from daily PR data to classify physical fatigue are mainly related to pain and problems in performing daily activities. For mental fatigue, in addition to pain and problems PR features, depressed mood resulted highly relevant. This suggests that physical fatigue is mainly associated with physical exhaustion, while mental fatigue is more like a psychobiological state (30, 47). Observations on classification results for the multi-class classification of daily tiredness show that, again features from both PR and Fitbit are among the top 20 important predictors of daily tiredness. The best XGBoost hyperparameters for this configuration are learning rate of 0.05, a maximum depth of 2 and number of threes of 100. However, the top 5 features comprises only of PR features. This could possibly be related to the moderate association (Goodman Kruskal τ of around 0.2) between the daily tiredness label and the other PR features.

When comparing the top predictive Fitbit features for the three fatigue types [PhF, MF and daily tiredness (T)], we observe that the resting heart rate is common in predicting all three fatigue types. Sleep features from Fitbit are more relevant in predicting PhF and daily tiredness and have less importance in predicting MF. The step features related to physical activity have similar importance in predicting all three fatigue types.

We further tested the performance of the classification method using only the top 20 features identified by XGBoost. We noticed that for all the three classification tasks, similar performance were obtained compared to when using all the features in the task-specific best approach. This shows the possibility of using a simplified model in real applications leveraging only the top 20 features in input to XGBoost. Additional analysis, not reported in this manuscript, was performed including reported daily tiredness as features for the recognition of weekly mental and physical fatigue. Results showed an improvement of 2–3 percentage point in balance accuracy compared to when this feature was not added. Future work could also focus on the inclusion of daily tiredness, as automatically derived from a multi-class model, to improve weekly fatigue recognition. Other classifiers i.e., logistic regression, support vector machine and random forest were tested, however they were outperformed by XGBoost. Additional future research following our initial exploratory approach, could extend the prediction tasks to regression or multi-class and multi-label classification for finer discrimination of different levels of weekly reported fatigue.

A unique feature of our study is that it included the participants in their real-life environment. However, several limitations must be recognized including no formal inclusion/exclusion criteria related to fatigue level, specific medications, or clinically-assessed disease activity for the SjS and SLE participant groups. Furthermore, participants in our study are mostly of white race, leading to a potential bias in the results. A downside is the smaller sample size (68 HV, 57 SjS and 58 SLE participants) used for the analysis, resulting from the exclusion of data from 113 participants with low data quality. Moreover, data points from participants with low adherence were also excluded from the analysis. This resulted in an imbalance between the participant groups used for the analysis with more HV than disease groups (SjS and SLE). Future work could potentially expand the adherence analysis by including exploration of additional behavioural patterns and the influence of contextual information (e.g., day of the week, hour of the day). Another limitation of our work consists in being potentially affected by variability in participants’ Fitbit device model and firmware version. The only device-specific inclusion criteria was the requirement of minute-level data that implicitly excluded older version of Fitbit. In this study, we did not perform additional validation of the consumer-grade wearable device (Fitbit) parameters. We instead relied on previous work on validating the main Fitbit parameters (48–50). We believe the results presented in our exploratory study could be used to inform future confirmatory work. Yet additional validation should be done before applying our method to clinical practice.

In our current modeling strategy, which we refer to as *population model*, data of participants from both HV and disease groups were used concurrently during training. Even though the type of diagnosis was used as a feature in the models, cohort-specific patterns might not be well-captured by the model, limiting its performance capability. In an initial attempt, cohort-specific models were trained using an incremental weeks strategy, where data of subsequent weeks were incrementally added to the training set to classify fatigue levels of the last week. Using this strategy, even if participant-dependent, we noticed an increment in performance compared to when using a population model, especially for SjS and SLE participants and for the recognition of non-fatigue weeks. In this study, data were collected over a period of 1 month. Longer studies would need to be considered to better understand and predict the phenomenon of chronic fatigue and fatigue related to chronic illness. Future work could further explore this aspect focusing, for instance, on fine-tuning population models with cohort-specific data. For instance, the population model trained on healthy volunteers and patients from an observational study, could be adapted to a cohort from a new clinical trial, leveraging previous knowledge but also refining to the new cohort characteristics. Both binary and multi-class classification analyses help to make practical recommendations for the clinical trials and support digital biomarker strategy for fatigue. Use of multi-sensor wearable device to measure vital signs and physical activity along with collecting frequent ePROs (e.g., on the same day as wearable data) should be considered in clinical trials. This approach would enhance research on quantitative measurement of fatigue and facilitate development of therapeutic strategies to address pathological fatigue (e.g., in SLE and SjS). Furthermore, machine-learning approach could be used to build a simplified model of fatigue and define a single, composite score to describe and validate a digital endpoint of fatigue as an objective measure. At the end of the study, we asked each participant through a final short survey, their level of satisfaction with their experience with this remote digital study. As a result, 52% of participants reported to be “extremely satisfied” and 39% “satisfied”. As also suggested by the aforementioned participant feedback, growing overall popularity and acceptance of using digital health technologies and wearable tools, combined with ease of their use in everyday life, creates opportunities for studying large cohorts of healthy individuals and in patient in future.

## Data Availability

The original contributions presented in the study are included in the article/**Supplementary Material**. The dataset analyzed for this study are available on Zenodo at https://zenodo.org/record/8018238. Further inquiries can be directed to the corresponding author.

## Footnotes

^1^ https://evidation.com/

^2^ https://fitbit.com

^3^ https://www.facit.org/

^4^The 13-item FACIT-Fatigue Score ranges between 0 and 52. A score lower than 30 indicates severe fatigue. The higher the score, the better the QoL (34).
