# Supplementary material for: Association of digital measures and self-reported fatigue: a remote observational study in healthy participants and participants with chronic inflammatory rheumatic disease
Source: Front Digit Health. 2023 Jun 22;5:1099456. doi: 10.3389/fdgth.2023.1099456 (PMC10324580; doi:10.3389/fdgth.2023.1099456)
Supplement: Supplementary file 1 [file Datasheet1.pdf]

SUPPLEMENTARY MATERIAL A

1 OVERVIEW OF STUDY POPULATION

The following plots supplement the overview of the study population on overall demographics (Table S1 and Figure S1), and participant-specific data, including demographics and medical history (Figures S2-S4).

Table S1. Study cohorts characteristics including age, sex and ethnic background. HV: healthy volunteers; SLE: systemic lupus erythematosus; SjS: Sjögren's syndrome; sd: standard deviation.

	HV	SLE	SjS	Total	Overall percentage
**Number of participants**	105	104	87	296	-
**Female**	79	103	87	269	90.9
**Male**	26	1	0	27	9.1
**Mean age ± sd**	43.9 ± 11.1	43.2 ± 11.2	43.9 ± 9.4	45.4 ± 11.0	
**Race**					
American Indian or Alaska Native	2	4	0	6	2.0
Asian	7	6	2	15	5.1
Black	14	10	8	32	10.8
Native Hawaiian Pacific Islander	1	0	0	0	0.3
White	84	87	76	247	83.4
Other	4	3	1	8	2.7
**Ethnicity**					
Non-Hispanic Latino	94	92	83	269	90.9
Hispanic Latino	11	12	4	27	9.1

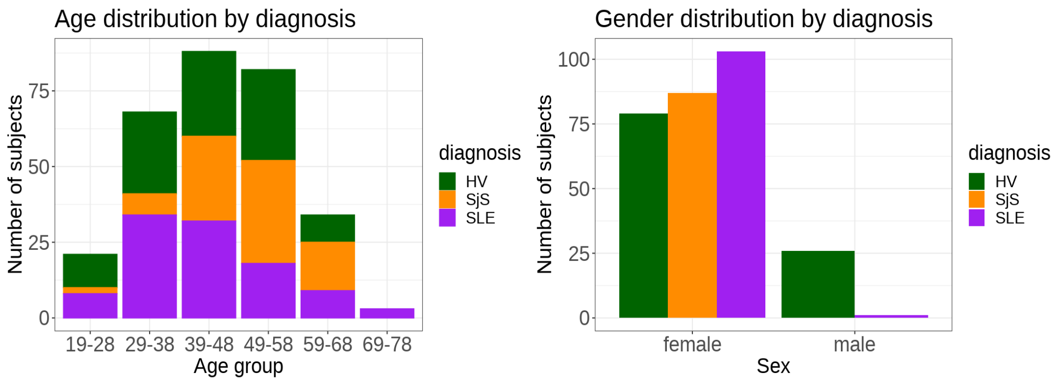

Figure S1: Distribution of number of participants for age groups and sex stratified by participants groups.

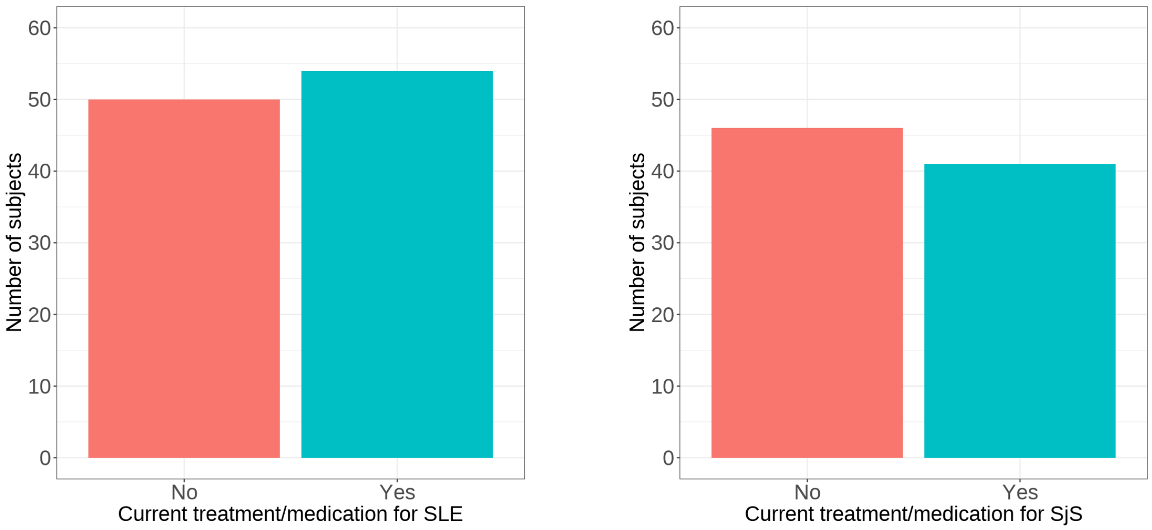

(a) (b)

Figure S2: Distribution of number of participants for current treatment/medication during survey for a) SLE and b) SjS.

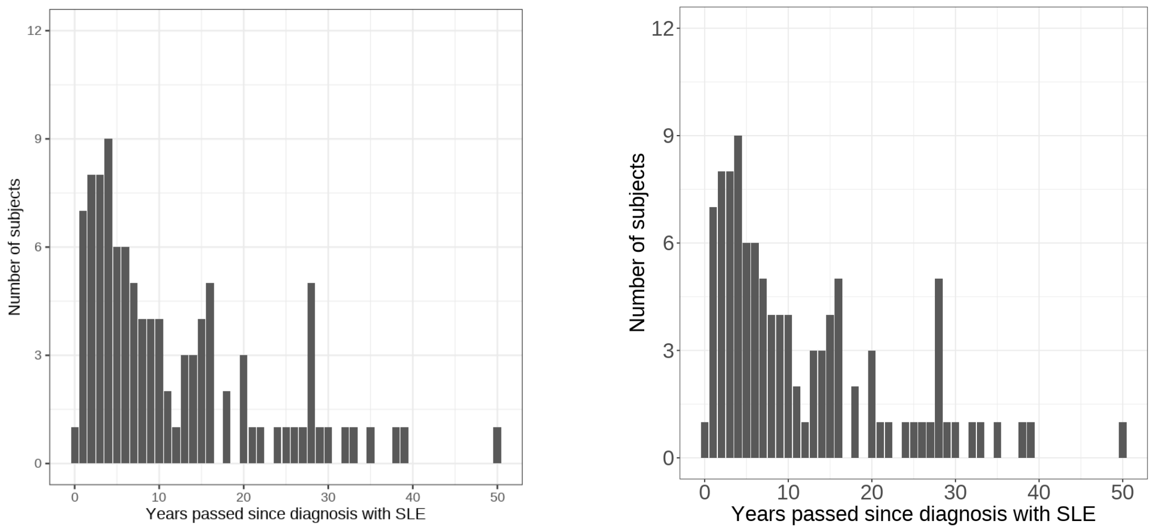

(a) (b)

Figure S3: Distribution plots showing number of years passed since participants were diagnosed with a) SLE and b) SjS.

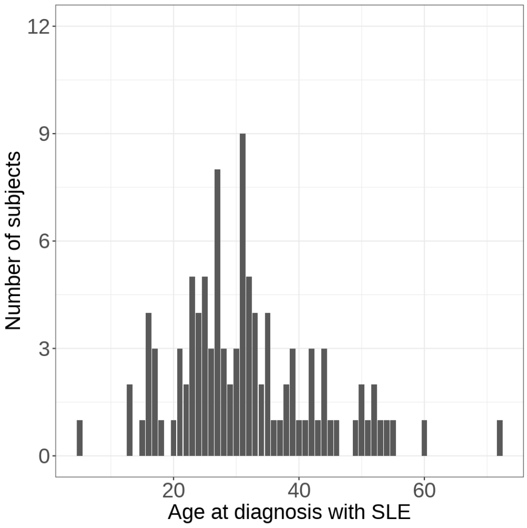

(a)

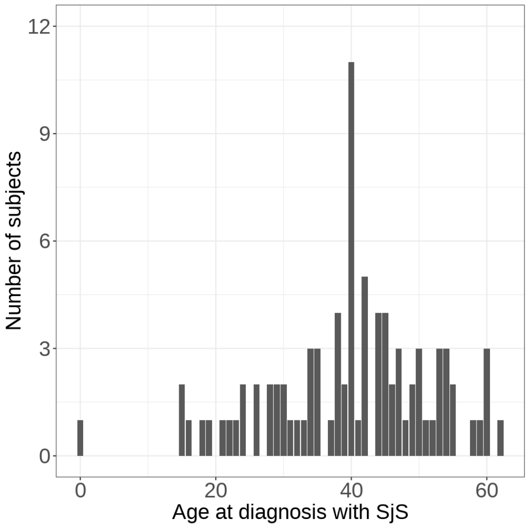

(b)

Figure S4: Distribution plots showing age at diagnosis for a) SLE participants and b) SjS participants.

2 STUDY ADHERENCE

A total of 296 participants were enrolled in the study, with only 207 of which worn the fitbit device during the study. The total number of days available for the fitbit users before data cleaning is 6992. To ensure data quality, we discarded days in which the device was worn for less than 60% of the time. Fig.S6 shows the distribution of daily wear-time per participant. The majority of participants were highly compliant, i.e. were wearing the device for most of the time. Only three participants never worn the device for more than 60% of the time in any day. These three participants were discarded from our analysis. Fitbit data of a total of 766 days were removed after data pre-processing and following the wear time thresholds and data from a total of 204 participants were kept for the analysis. Fig.S7 shows the wear time across days, it is possible to observe that less compliant participants tended to wear the device less frequently compared to the more compliant ones. No clear temporal pattern in changes of wear-time during the course of the study was observed.

Regarding participants reported data, 293 participants filled in at least one daily list of questions and 272 participants have at least 15 overall days of completed list of questions. The average number of list of questions answered by participants over the study is 26 (median 29 IQR [25:30]). Fig.S5 shows the distribution of compliance in filling in daily list of questions across the study days for all the participants. We did not observe consistent patterns in missing entries for this data modality. The synchronization of Fitbit data with the weekly and daily list of questions, together with removal of 503 observations with missing values (either a specific Fitbit feature or a specific question), lead to a total of 3950 observations (days) from 183 participants.

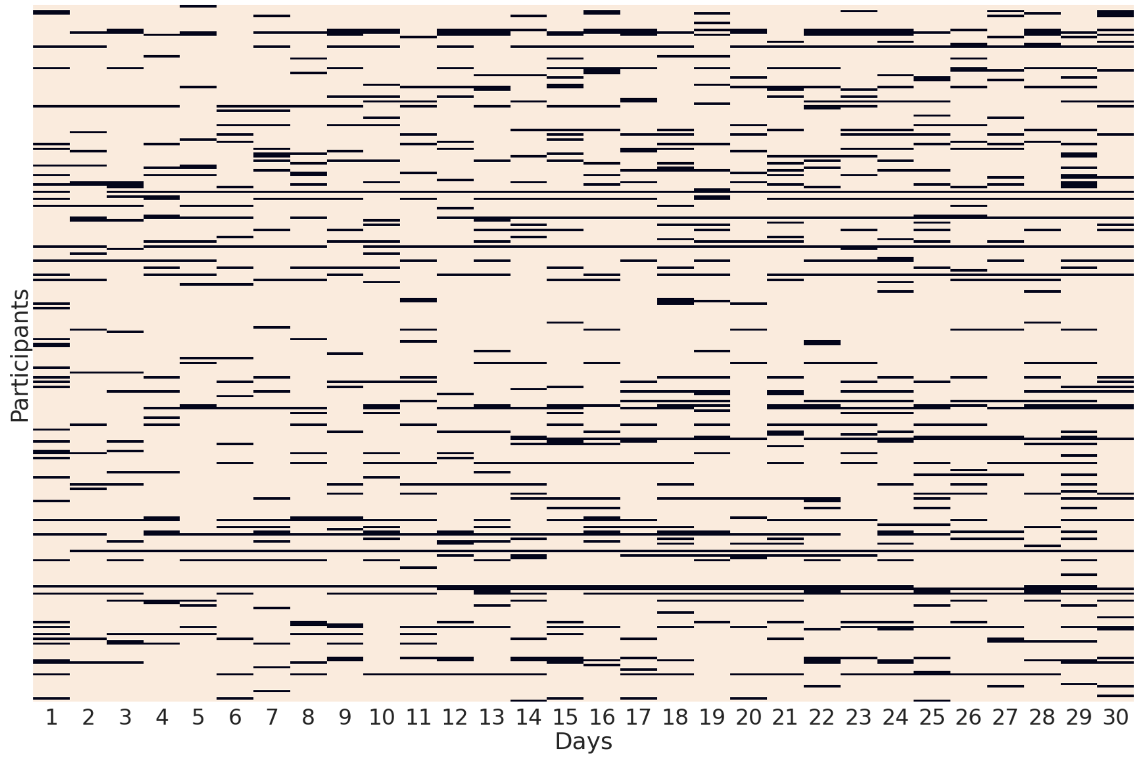

Figure S5: Participants' adherence in completing daily survey. The heatmap shows study days (x-axis) in which participants (y-axis) filled in (light color) or didn't fill in (dark color) the daily questions across adjusted study days.

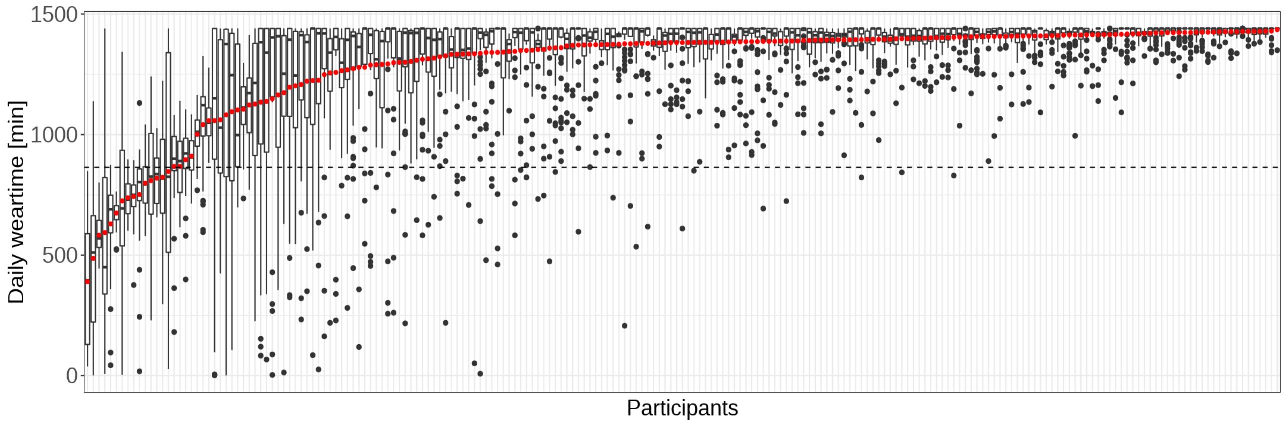

Figure S6: Participants' adherence in wearing Fitbit. Daily wear time in minutes is reported per participant. Participants are sorted in ascending order by their study average daily wear time. The dashed line represents the threshold of 60% (864 minutes) used in this work to remove days with low wear time.

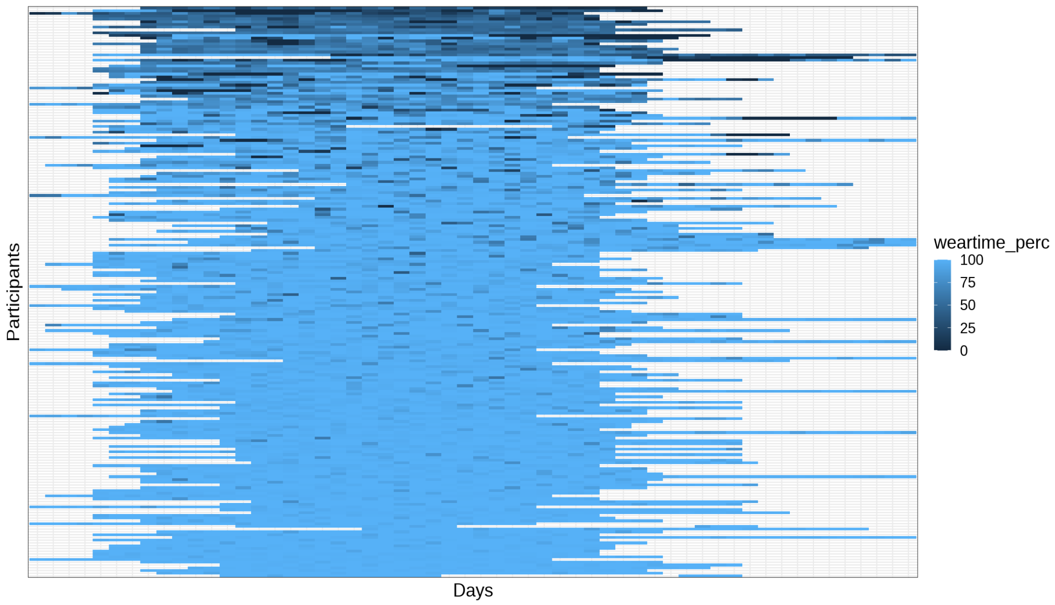

Figure S7: Participants' adherence in wearing Fitbit over days. The heatmap shows the percentage of daily wear time per participant across calendar days. Lighter colors show higher percentage of wear time while darker colors lower percentage of wear time. Participants are sorted in ascending order by their average daily wear time.

3 DETAILS OF APP-BASED SURVEY DATA

Sections 3.1 and 3.2 list questions and multiple choice answers of the weekly and daily survey. Full list of final PR features used for the analysis is given in S2. Results of the factor analysis of mixed data (FAMD) of PR features are summarized in the Figure S19.

In Figure S8, distributions of daily survey answers on daily rating, usual activities, pain, sleep, mood and tiredness are illustrated per participant group (HV, SLE, SjS). Participants answered to the daily questions mostly in the morning, specifically 74% of the surveys were reported in the morning (7-11am), 17% in the afternoon (12-6pm) and 8% later in the day (7-11pm). Figure S9 reports the association map between the daily features.

FACIT-Fatigue scores are plotted in FigureS10. The association between FACIT-Fatigue score and PR are showed in S12, and weekly mental and physical fatigue scores in S11. Weekly survey data on mental and physical fatigue are summarized in Figures S13 and S15, with stratification for diagnosis and medication.

3.1 Daily List of Questions

[General - day_rating] How was your day yesterday?

1.Good
2.Average
3.Bad

[Usual activities - $usual\_activities\_problems$] Did you have problems in doing your usual activities (e.g. work, study, housework, family or leisure activities) yesterday?

1.Not at all
2.Slight problems
3.Moderate problems
4.Severe problems
5.Extreme problems

[Usual activities - $problem\_frequency$] How often did you have problems in doing your usual activities (e.g. work, study, housework, family or leisure activities) yesterday?

1.Not at all
2.Some of the time
3.Most of the time
4.All of the time

[Pain - $pain\_yesterday$] Did you experience any pain yesterday?

1.Not at all
2.Slight pain
3.Moderate pain
4.Severe pain
5.Extreme pain

[Pain - $pain\_frequency$] How often did you experience pain yesterday?

1.Not at all
2.Some of the time
3.Most of the time
4.All of the time

[Sleep - $sleep\_yesterday$] Did you need to sleep during the day yesterday?

1.Not at all
2.Yes, once
3.Yes, multiple times

[Mood - $depressed\_yesterday$] I felt depressed yesterday:

1.Not at all
2.Slightly
3.Moderately
4.Severely
5.Extremely

[Mood - $depressed\_frequency$] Thinking about yesterday, how often were you bothered by feeling down, depressed or hopeless?

1.Not at all
2.Some of the time

3.Most of the time
4.All of the time

[Fatigue - $tired\_yesterday$] Did you feel tired yesterday?

1.Not at all
2.A little bit
3.Somewhat
4.Quite a bit
5.Very much

3.2 Weekly questions

[Mood] In the last week I felt depressed:

1.Not at all
2.Slightly
3.Moderately
4.Severely
5.Extremely

[Mood] Over the last week, how often were you bothered by feeling down, depressed or hopeless?

1.Not at all
2.Some of the time
3.Most of the time
4.All of the time

[Fatigue] Physically, in the last week how often did you feel exhausted?

1.Never
2.Sometimes
3.Regularly
4.Often
5.Always

[Fatigue] Mentally, in the last week how often did you feel exhausted?

1.Never
2.Sometimes
3.Regularly
4.Often
5.Always

Table S2. List of PR features used for further analysis, the $*$ indicates features selected by FAMD.

Feature	Description
usual_activities_problems*	Did you have problems in doing your usual activities (e.g. work, study, housework, family or leisure activities) yesterday?
pain_yesterday*	Did you experience pain yesterday?
problem_frequency*	How often did you have problems in doing your usual activities (e.g. work, study, housework, family or leisure activities) yesterday?
depressed_yesterday*	I felt depressed yesterday:
depressed_frequency*	Thinking about yesterday, how often were you bothered by feeling down, depressed or hopeless?
pain_frequency*	How often did you feel pain yesterday?
day_rating	How was your day yesterday?
sleep_yesterday	Did you need to sleep during the day yesterday?

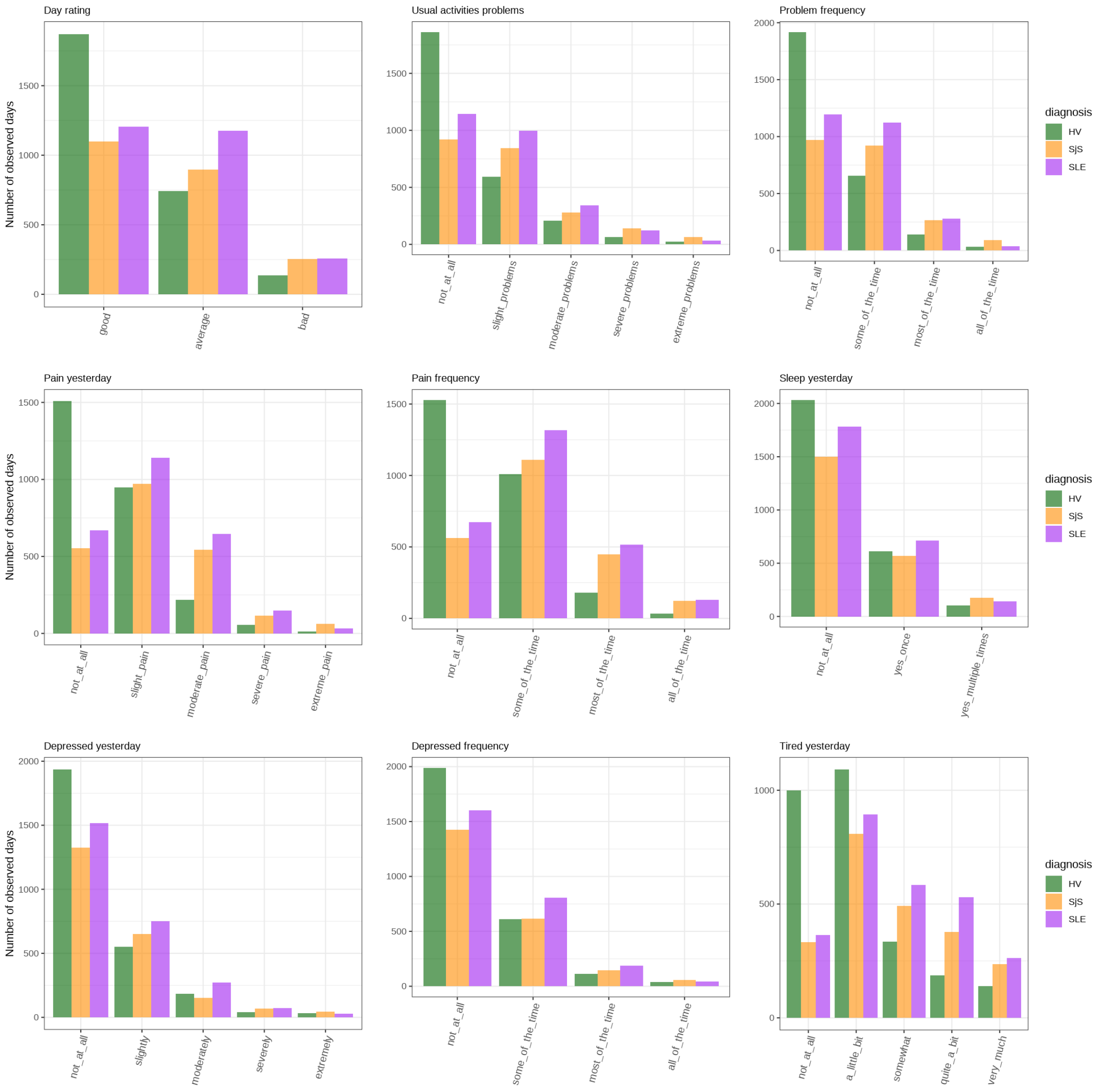

Figure S8: Distribution of survey answers for each question from the daily questions domain stratified by participant groups.

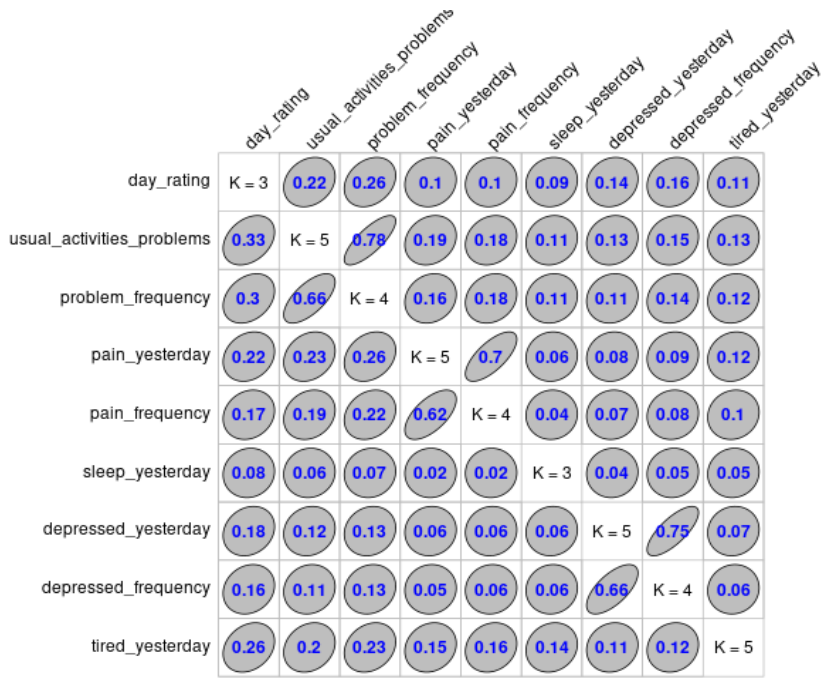

Figure S9: Association map showing the Goodman and Kruskal's τ to test correlation between categorical PRs. Diagonal element K refers to the number of unique levels (possible answers) per question in the survey.

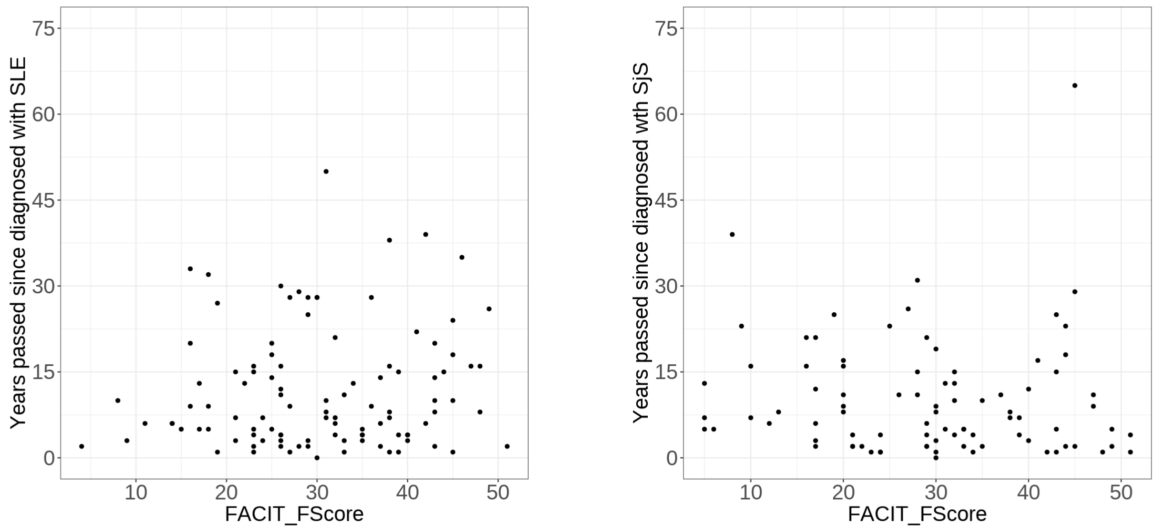

(a) (b)

Figure S10: Distribution of FACIT-Fatigue score with respect to the number of years passed since the participant was diagnosed with (a) SLE and (b) SjS.

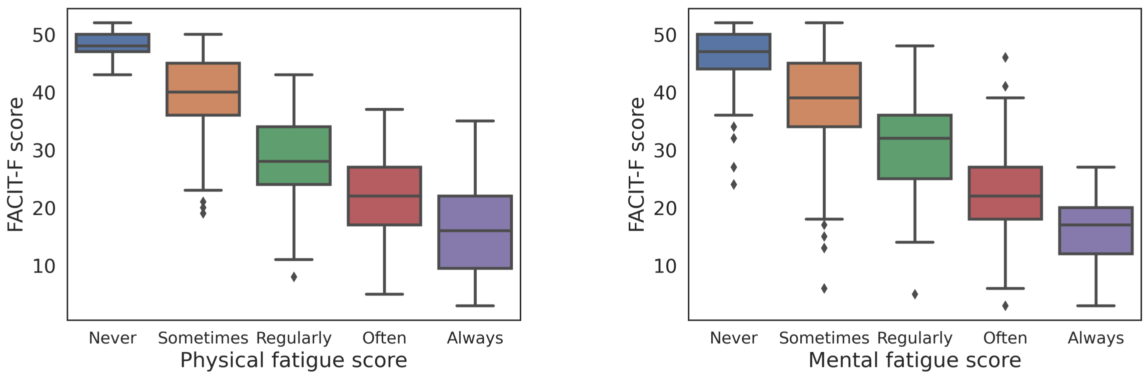

Figure S11: Relation of different levels of physical and mental fatigue with FACIT-F score.

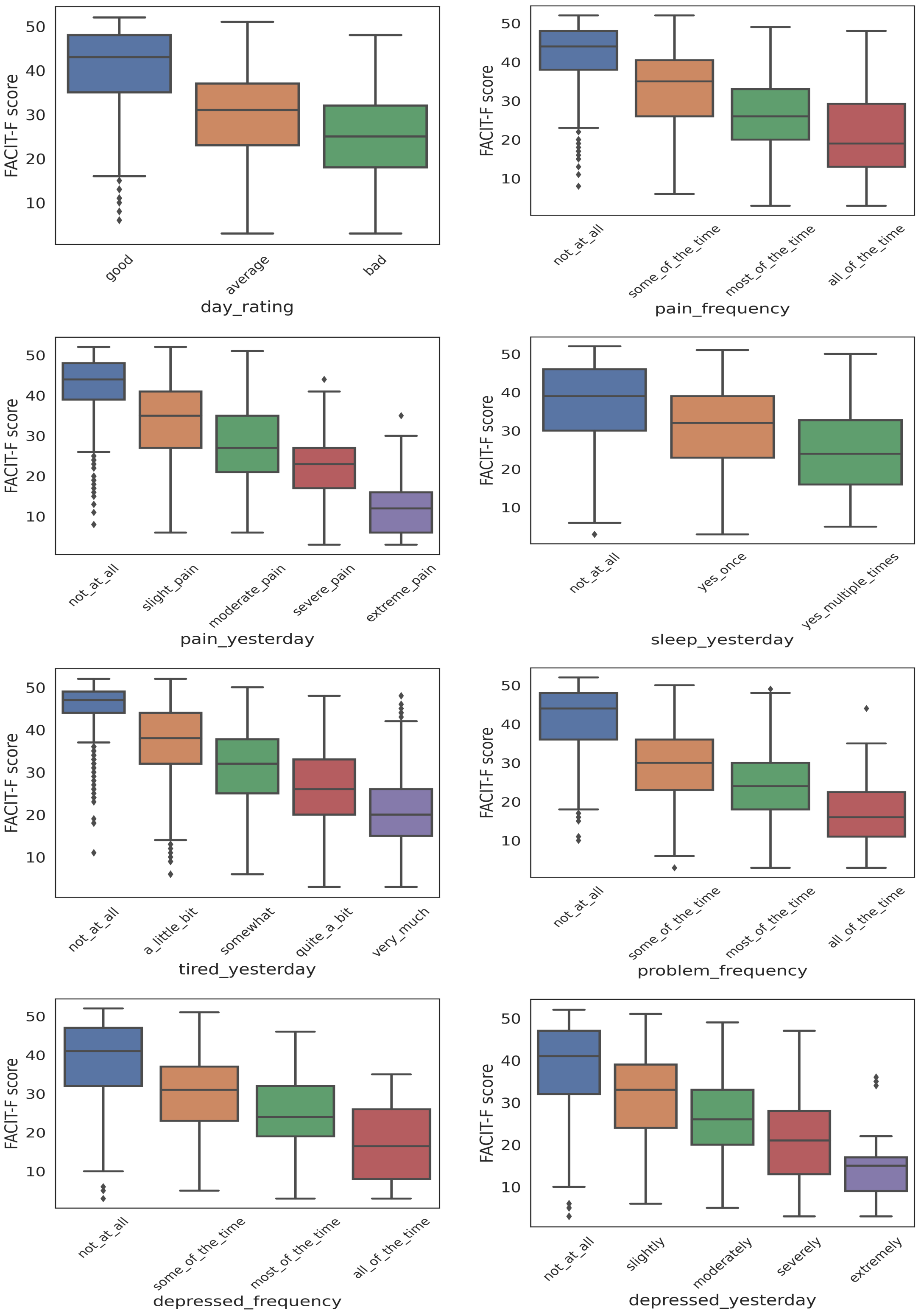

Figure S12: Relation between FACIT-F and daily questions.

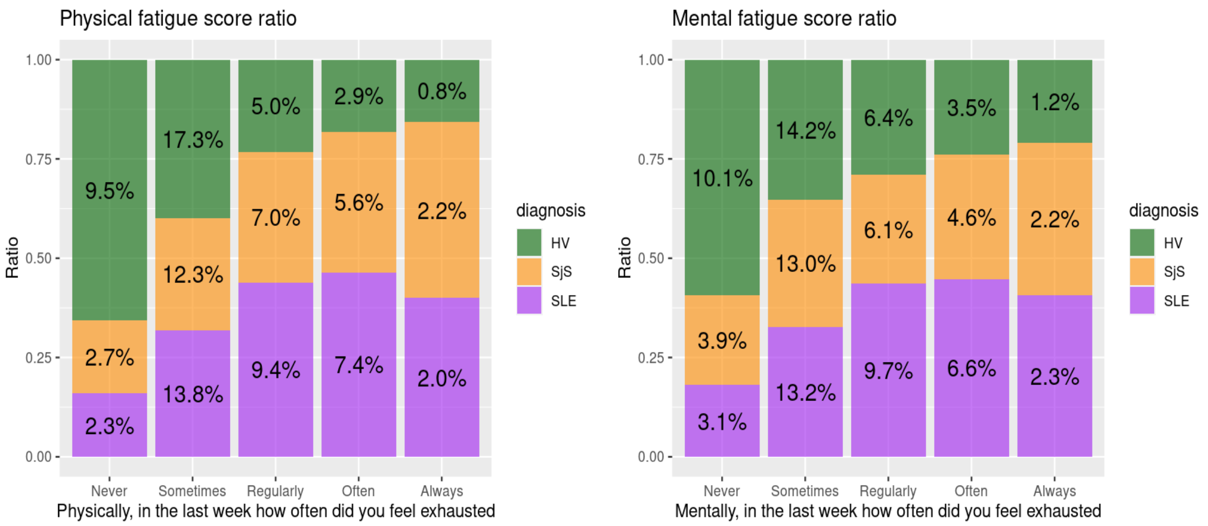

Figure S13: Ratio of different levels of physical and mental fatigue stratified by participant groups.

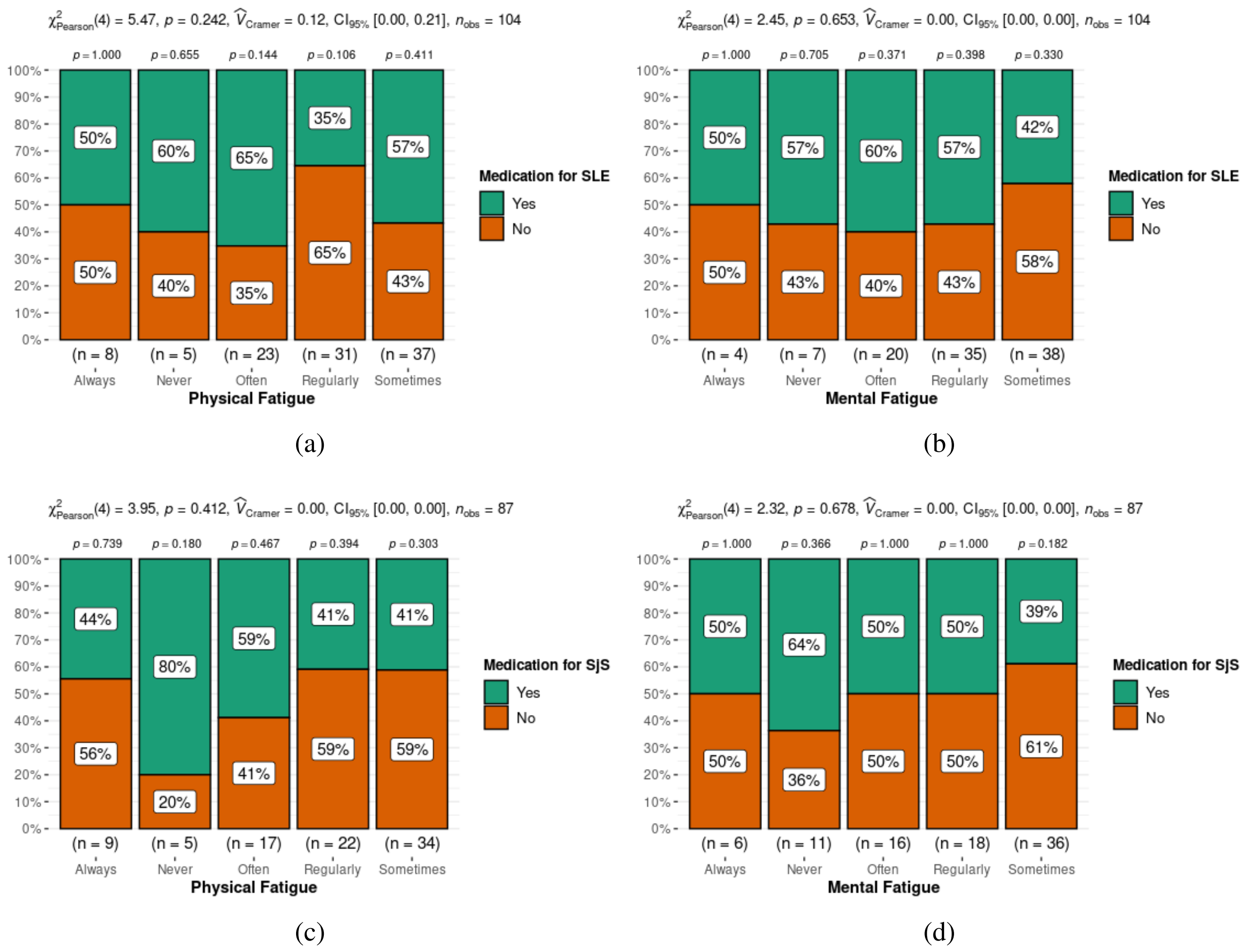

Figure S14: Statistical analysis showing no significant association between treatment and fatigue levels. The values on top of each plot represent the statistical details and help us to understand the relationship between medication and fatigue levels: Pearson's test of independence, p-value, confidence intervals and number of observations. The values inside the plot tells us the percentage of participants taking or not taking any medication for SLE and SjS complaining of different levels of physical and mental fatigue. a) Association of treatment or medication for SLE with physical fatigue levels. b) Association of treatment or medication for SLE with mental fatigue levels. c) Association of treatment or medication for SjS with physical fatigue levels. d) Association of treatment or medication for SjS with mental fatigue levels.

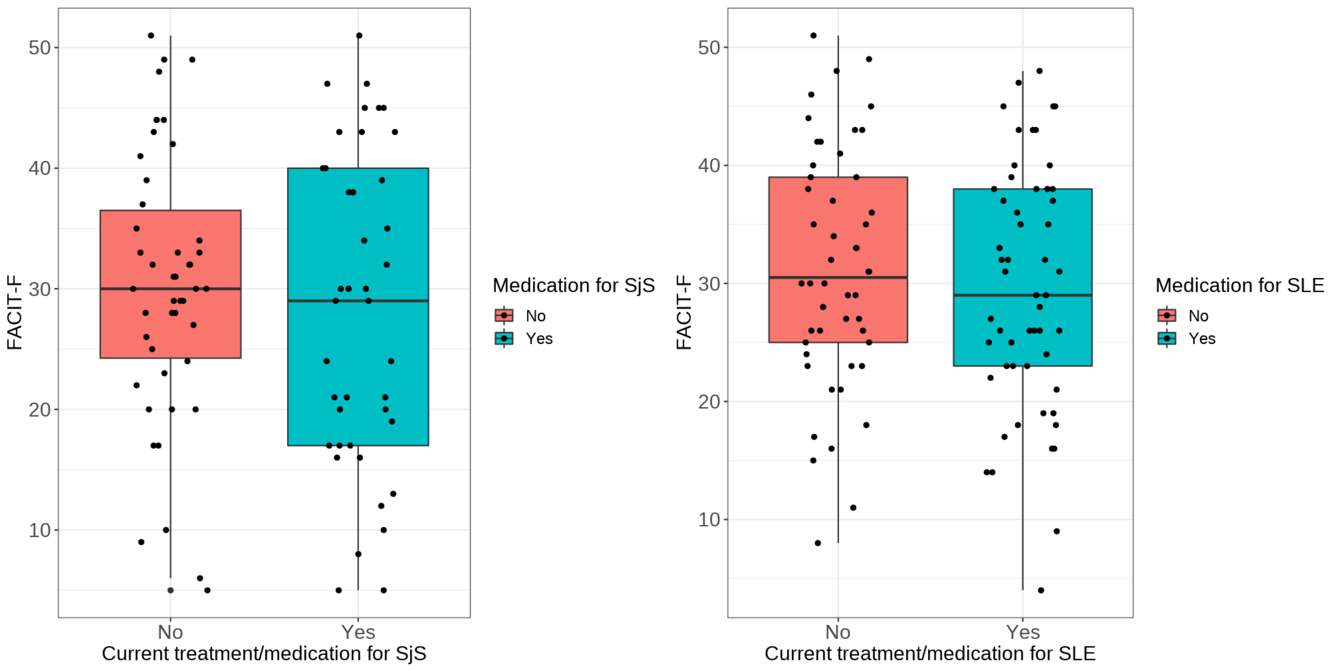

(a)

(b)

Figure S15: FACIT-F score distribution between participants taking or not any medication for a) SjS b) and SLE. No statistical difference (p-value >0.05 from Mann Whitney U test), was observed between treatment and FACIT-F score for both SLE and SjS participants.
