# Supplementary material for: Association of digital measures and self-reported fatigue: a remote observational study in healthy participants and participants with chronic inflammatory rheumatic disease
Source: Front Digit Health. 2023 Jun 22;5:1099456. doi: 10.3389/fdgth.2023.1099456 (PMC10324580; doi:10.3389/fdgth.2023.1099456)
Supplement: Supplementary file 2 [file Datasheet2.pdf]

SUPPLEMENTARY MATERIAL B

4 DETAILS OF DAILY FITBIT DATA

In this section, the full list of collected daily Fitbit features is shown in Tables S3, S4 and S5 for heart rate (HR), steps, and sleep respectively. Features were obtained from the Evidation Health platform and from the Fitbit device directly.

Table S3. Full list of **heart rate (HR)** features from Fitbit data. Features highlighted in gray cells are the ones used for the classification analysis, the $*$ symbol indicates features selected by the PCA. The symbol $\diamond$ indicates features obtained from the Fitbit directly.

**Feature**	**Description**
heart_rate__asleep__max	Maximum HR while ”asleep” sleep pattern
heart_rate__asleep__mean*	Mean HR while ”aslep” sleep pattern
heart_rate__asleep__stddev*	Standard deviation of HR while ”asleep” sleep pattern
heart_rate__autocorr10min*	Correlation between each HR value and the value measured 10 minutes before it
heart_rate__autocorr1min*	Correlation between each HR value and the value measured 1 minute before it
heart_rate__autocorr2min*	Correlation between each HR value and the value measured 2 minutes before it
heart_rate__autocorr3min*	Correlation between each HR value and the value measured 3 minutes before it
heart_rate__autocorr60min	Correlation between each HR value and the value measured 60 minutes before it
heart_rate__awake__max*	Maximum HR while awake
heart_rate__awake__mean*	Mean HR while awake
heart_rate__awake__stddev*	Standard deviation of HR while awake
heart_rate__count	Number of minutes with HR $>$ 0
heart_rate__high_150_perc_resting_frac	Percent of minutes with HR larger than 1.5x resting HR for the day
heart_rate__high_200_perc_resting_frac	Percent of minutes with HR larger than 2.0x resting HR for the day
heart_rate__max*	Maximum HR
heart_rate__mean*	Mean HR
heart_rate__median*	Median HR
heart_rate__min*	Minimum HR
heart_rate__not_moving__max	Maximum HR while not moving
heart_rate__not_moving__mean*	Mean HR while not moving
heart_rate__not_moving__stddev*	Standard deviation of HR while not moving
heart_rate__peaks	Number of relative HR peaks in a day
heart_rate__peaks_gt_125_perc_median	Number of peaks larger than 1.25x the median HR for the day
heart_rate__peaks_gt_150_perc_median	Number of peaks larger than 1.5x the median HR for the day
heart_rate__peaks_gt_200_perc_median	Number of peaks larger than 2.0x the median HR for the day
heart_rate__perc_25th*	25th percentile of HR
heart_rate__perc_50th*	50th percentile of HR
heart_rate__perc_5th*	5th percentile of HR
heart_rate__perc_75th*	75th percentile of HR
heart_rate__perc_95th*	95th percentile of HR
heart_rate__quant_95th_to_resting_ratic	Ratio of 95th percentile HR in a day to the day’s resting HR
heart_rate__resting_heart_rate*$\diamond$	Resting HR
heart_rate__stddev*	Standard deviation of HR
heart_rate__mean__mean	Mean of heart_rate__mean over each month (Average monthly heart rate)
heart_rate__resting_heart_rate__mean	Mean of heart_rate__resting_heart_rate over each month (Average monthly resting heart rate)
heart_rate__mean__max	Maximum of heart_rate__mean over each month (Max monthly heart rate)
heart_rate__resting_heart_rate__max	Maximum of heart_rate__resting_heart_rate over each month (Max monthly resting heart rate)
heart_rate__mean__min	Minimum of heart_rate__mean over each month (Min monthly heart rate)
heart_rate__resting_heart_rate__min	Minimum of heart_rate__resting_heart_rate over each month (Min monthly resting heart rate)
heart_rate__minute_level_str	Raw minute level heart rate string

Table S4. Full list of **steps** features form Fitbit. Features highlighted in gray cells are the ones used for the classification analysis, the $*$ symbol indicates features selected by the PCA. The symbol $\diamond$ indicates features obtained from the Fitbit directly.

Feature	Description
steps__count*	Number of minutes with > 0 steps
steps__dec_time__max*	Decimal time of max steps minute taken of the day
steps__dec_time__min*	Decimal time of min steps minute taken of the day
steps__dec_time_max_rolling_6__first*	Decimal time of max of 6-minute rolling steps sum
steps__first_step_dec_time*	Decimal time of first step taken of the day
steps__gt10_nonzero_frac	Fraction of minutes with > 10 steps out of non-zero
steps__gt100_nonzero_frac*	Fraction of minutes with > 100 steps out of non-zero
steps__gt140_nonzero_frac	Proportion of minutes with > 140 steps among all minutes with non-zero steps
steps__gt50_nonzero_frac*	Proportion of minutes with > 50 steps among all minutes with non-zero steps
steps__last_step_dec_time*	Decimal time of last step taken of the day
steps__light_activity__sum	Number of minutes of light activity (between 50 and 100 steps per minute)
steps__max_rolling_1	Max of 1-minute rolling sum
steps__max_rolling_10*	Max of 10-minute rolling sum
steps__max_rolling_3*	Max of 3-minute rolling sum
steps__max_rolling_30*	Max of 30-minute rolling sum
steps__max_rolling_6*	Max of 6-minute rolling sum
steps__max_rolling_60*	Max of 60-minute rolling sum
steps__max_rolling_dec_time_1	Decimal time of max of 1-minute rolling sum
steps__max_rolling_dec_time_10*	Decimal time of max of 10-minute rolling sum
steps__max_rolling_dec_time_3	Decimal time of max of 3-minute rolling sum
steps__max_rolling_dec_time_30	Decimal time of max of 30-minute rolling sum
steps__max_rolling_dec_time_6*	Decimal time of max of 6-minute rolling sum
steps__max_rolling_dec_time_60	Decimal time of max of 60-minute rolling sum
steps__midday_steps_dec_time	Decimal time at which steps taken equals steps to be taken
steps__mvpa__sum	Number of minutes of moderate or vigorous physical activity (> 100 steps per minute)
steps__non_zero_frac*	Fraction of minutes with non-zero steps
steps__nonzero_50quantile	50th quantile of non-zero steps distribution
steps__nonzero_5quantile	5th quantile of non-zero steps distribution
steps__nonzero_95quantile*	95th quantile of non-zero steps distribution
steps__not_moving__sum	Number of steps where not moving
steps__rolling_6_sum__max*	Maximum 6-minute rolling steps sum
steps__sedentary__sum*	Number of minutes when step count is less than 50 steps
steps__streaks__countDistinct*	Number of consecutive non-zero step streaks
steps__sum*	Number of steps
steps__sum__mean	Mean of steps__sum over each month (Average monthly steps)
steps__sum__max	Maximum of steps__sum over each month (Max monthly steps)
steps__sum__min	Minimum of steps__sum over each month (Min monthly steps)
steps__sum__sum	Sum of steps__sum over each month (Total monthly steps)
steps__minute_level_str	Raw minute level steps string

Table S5. Full list of **sleep** features form Fitbit. Features highlighted in gray cells are the ones used for the classification analysis, the $*$ symbol indicates features selected by the PCA. The symbol $\diamond$ indicates features obtained from the Fitbit directly.

Feature	Description
sleep__asleep__mean*	Proportion of sleep minutes in ”asleep” sleep pattern
sleep__asleep__sum*	Minutes of ”asleep” sleep pattern
sleep__awake__mean*	Proportion of sleep minutes in ”restless” or ”awake” sleep patterns
sleep__awake__sum*	Minutes of ”restless” and ”awake” sleep patterns
sleep__awake_regions__countDistinct*	Count of distinct streaks where ”awake” or ”restless”
sleep__loop_main_asleep_frac*	Fraction of minutes during the main sleep spent in ”asleep” sleep pattern
sleep__loop_main_asleep_minutes*	Minutes in the main sleep spent in ”asleep” sleep pattern
sleep__loop_main_awake_frac*	Fraction of minutes during the main sleep spent in ”restless” or ”awake” sleep patterns
sleep__loop_main_awake_minutes*	Minutes in the main sleep spent in ”restless” or ”awake” sleep patterns
sleep__loop_main_awake_region_count*	Number of contiguous regions in ”restless” or ”awake” sleep patterns
sleep__loop_main_really_awake_frac*	Fraction of minutes during the main sleep spent in ”awake” sleep pattern
sleep__loop_main_really_awake_minutes*	Minutes in the main sleep spent in ”awake” sleep pattern
sleep__loop_main_really_awake_region_count	Number of contiguous regions in ”awake” sleep pattern
sleep__loop_main_to_wakeup_minutes	Minutes in bed after waking up
sleep__loop_main_until_sleep_minutes	Minutes in bed before fallling asleep
sleep__main_asleep_minutes*◇	Minutes asleep for only the main sleep
sleep__main_efficiency*◇	Sleep efficiency score during main sleep
sleep__main_in_bed_minutes*◇	Number of minutes in bed for only the main sleep of the day
sleep__main_start_minute◇	Minute of the day when the main sleep starts
sleep__main_start_time◇	Datetime of main sleep start
sleep__nap_count*◇	Number of naps
sleep__num_sleeps*◇	Number of sleeps (main sleep + naps) in a day
sleep__really_awake__mean	Proportion of sleep minutes in ”awake” sleep pattern
sleep__really_awake__sum*	Minutes of ”awake” sleep pattern
sleep__really_awake_regions__countDistinct	Count of contiguous regions of ”awake” sleep
sleep__sleeping__sum*	Minutes of ”asleep”; ”restless”; and ”awake” sleep patterns
sleep__total_asleep_minutes*◇	Minutes asleep
sleep__total_in_bed_minutes*◇	Minutes in bed
sleep__asleep__sum__mean	Mean of sleep__asleep__sum over each month (Average monthly sleep)
sleep__asleep__sum__max	Maximum of sleep__asleep__sum over each month (Max monthly sleep)
sleep__asleep__sum__min	Minimum of sleep__asleep__sum over each month (Min monthly sleep)
sleep__asleep__sum__sum	Sum of sleep__asleep__sum over each month (Total monthly sleep)
sleep__minute_level_str	Raw minute level sleep string

5 PRINCIPAL COMPONENT ANALYSIS

Dimensionality reduction was performed using Principal Component Analysis (PCA) on the numerical daily Fitbit features for each set independently. Features were normalized to have a standard deviation of 1 and a threshold of 80% was used for the cumulative percentage of total variance to select the top principal components (PCs). Factor analysis of mixed data (FAMD) was applied to the categorical daily PR features. We calculated the total contribution of each feature to the top PCs as the weighted sum of the eigenvalues (Es) of the top PCs by the contributions (Cs) of the features to each PCs divided by the sum of the eigenvalues. For example, the total contribution of feature f to the first two PCs (PC_1, PC_2) is:

$$total\_contribution\_PC_{1,2} = \frac{(C_1 \times E_1) + (C_2 \times E_2)}{E_1 + E_2}$$

Figures S5-S8 show the percentage of variance explained by each dimension to determine the top PCs and the total contribution of the features to top PCs. Features with total contribution higher than the expected average contribution (showed with red dashed line in the plots) were selected. To perform this analysis we used the R function *fviz_contrib* [1].

Results of the principal component analysis (PCA) of Fitbit features are summarized in Figures S16-S18. Correlation matrix of the PCA-filtered Fitibit features is shown in Figure S20. Distribution of all PCA-filtered Fitbit features across diagnosis are shown in FiguresS21-S31.

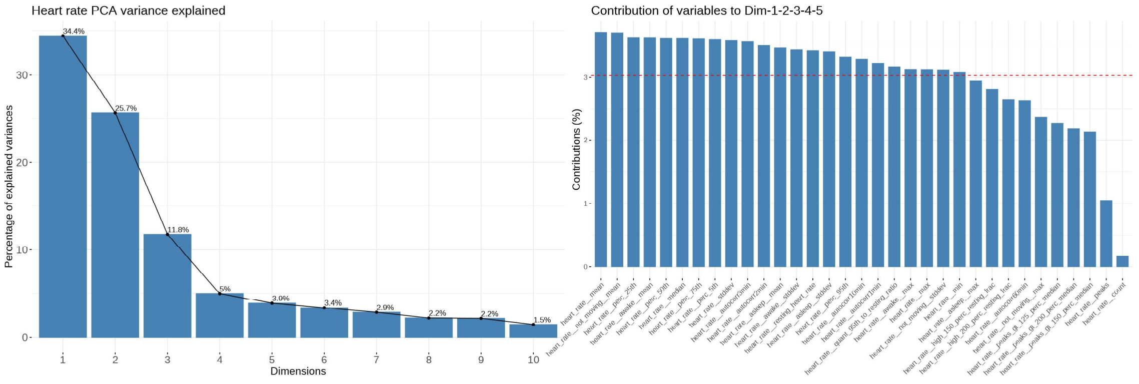

Figure S16: Percentage of variance explained by each dimension to determine the top PCs and contribution of HR features to top PCs.

[1] https://rdrr.io/cran/factoextra/man/fviz_contrib.html

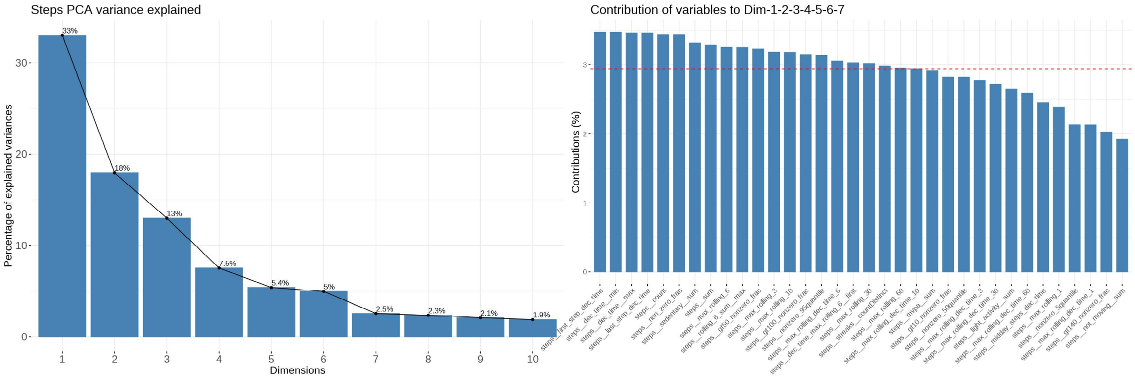

Figure S17: Percentage of variance explained by each dimension to determine the top PCs and contribution of step features to top PCs.

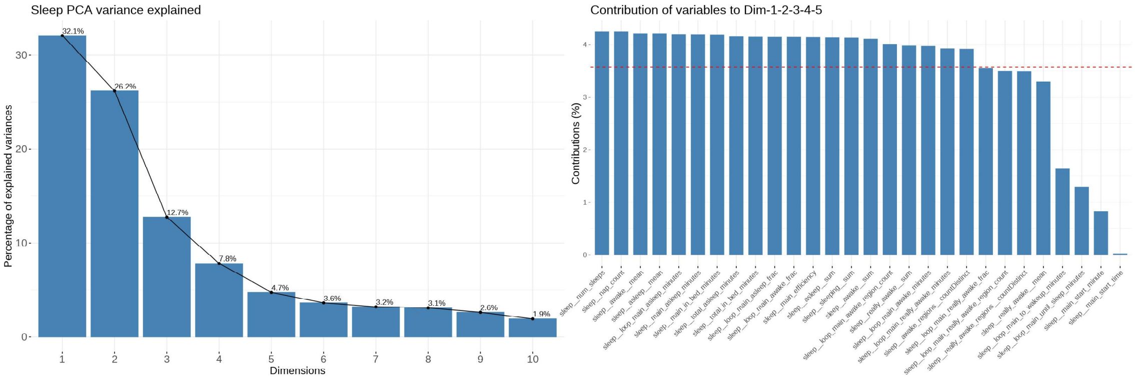

Figure S18: Percentage of variance explained by each dimension to determine the top PCs and contribution of sleep features to top PCs.

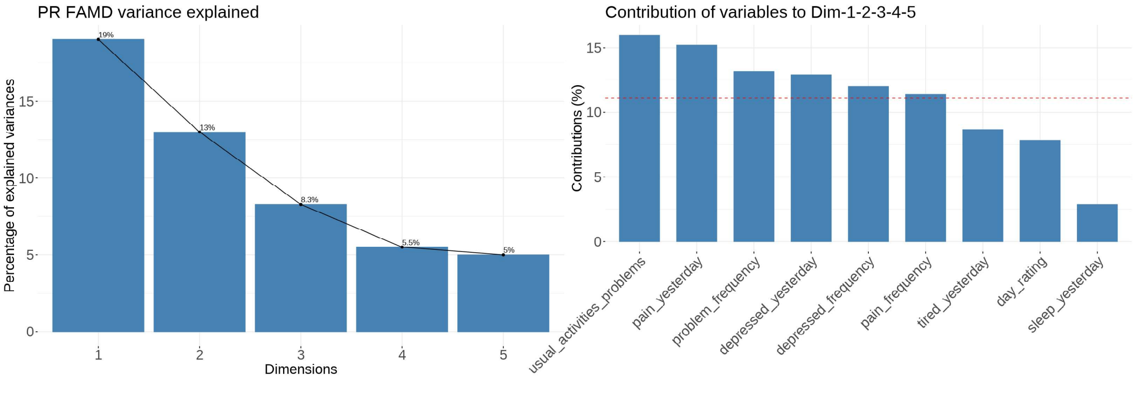

Figure S19: Percentage of variance explained by each dimension to determine the top PCs and contribution of PR features to top PCs.

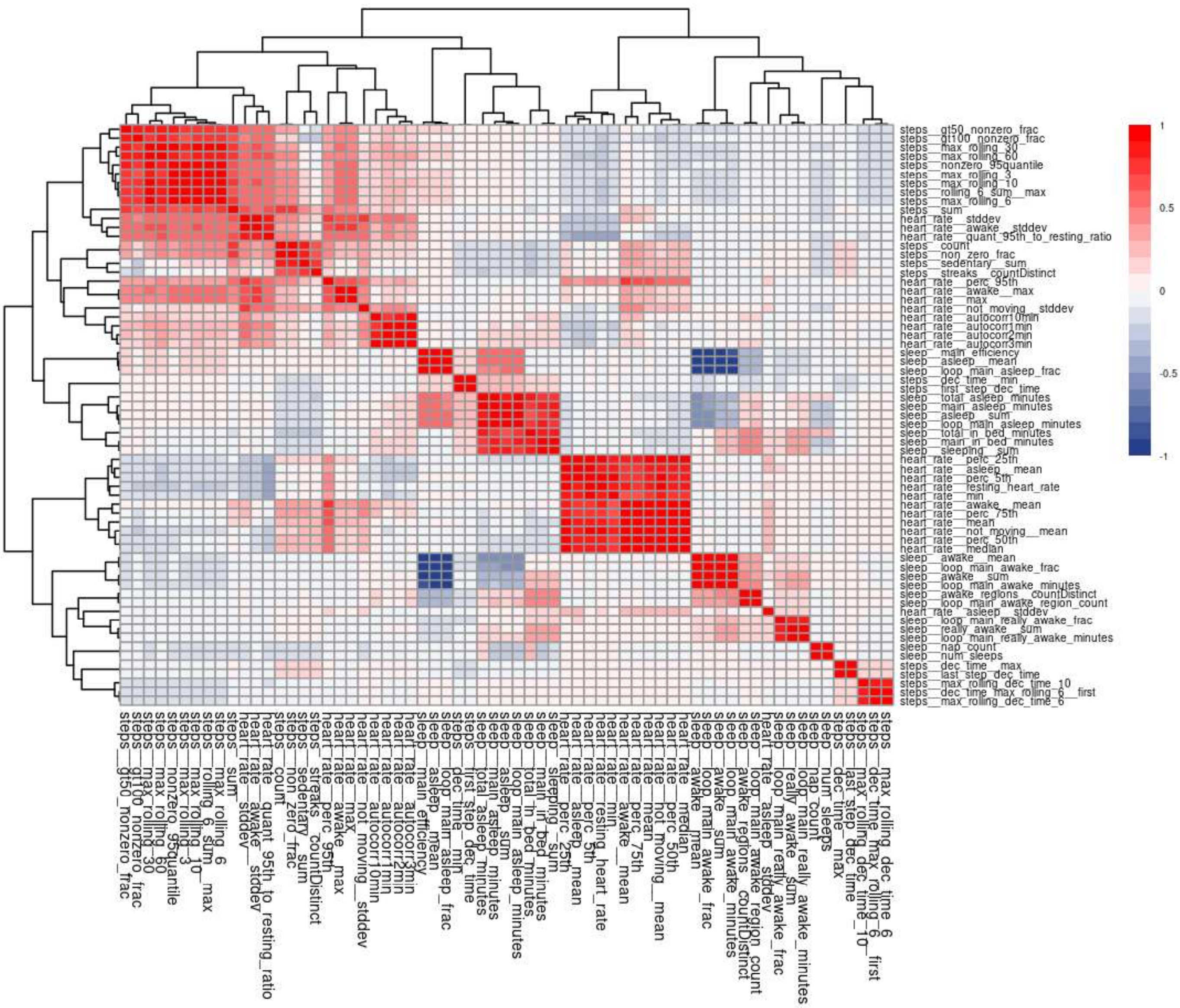

Figure S20: Pearson correlation heatmap of PCA-filtered Fitbit features.

6 FEATURES DISTRIBUTION DIFFERENCES IN THE THREE STUDIED GROUPS

Differences in Fitbit features were investigated in the three studied groups. Non-parametric Kruskal-Wallis one-way ANOVA test was used to identify significant differences. Dunn's test was used for post hoc pairwise analysis and Holm-Bonferroni method was used for adjusting p-values. Figures S21 - S31 show the overlay of violin and box plots for all 65 PCA-filtered Fitbit features. P-values resulting from the tests are reported on the figures.

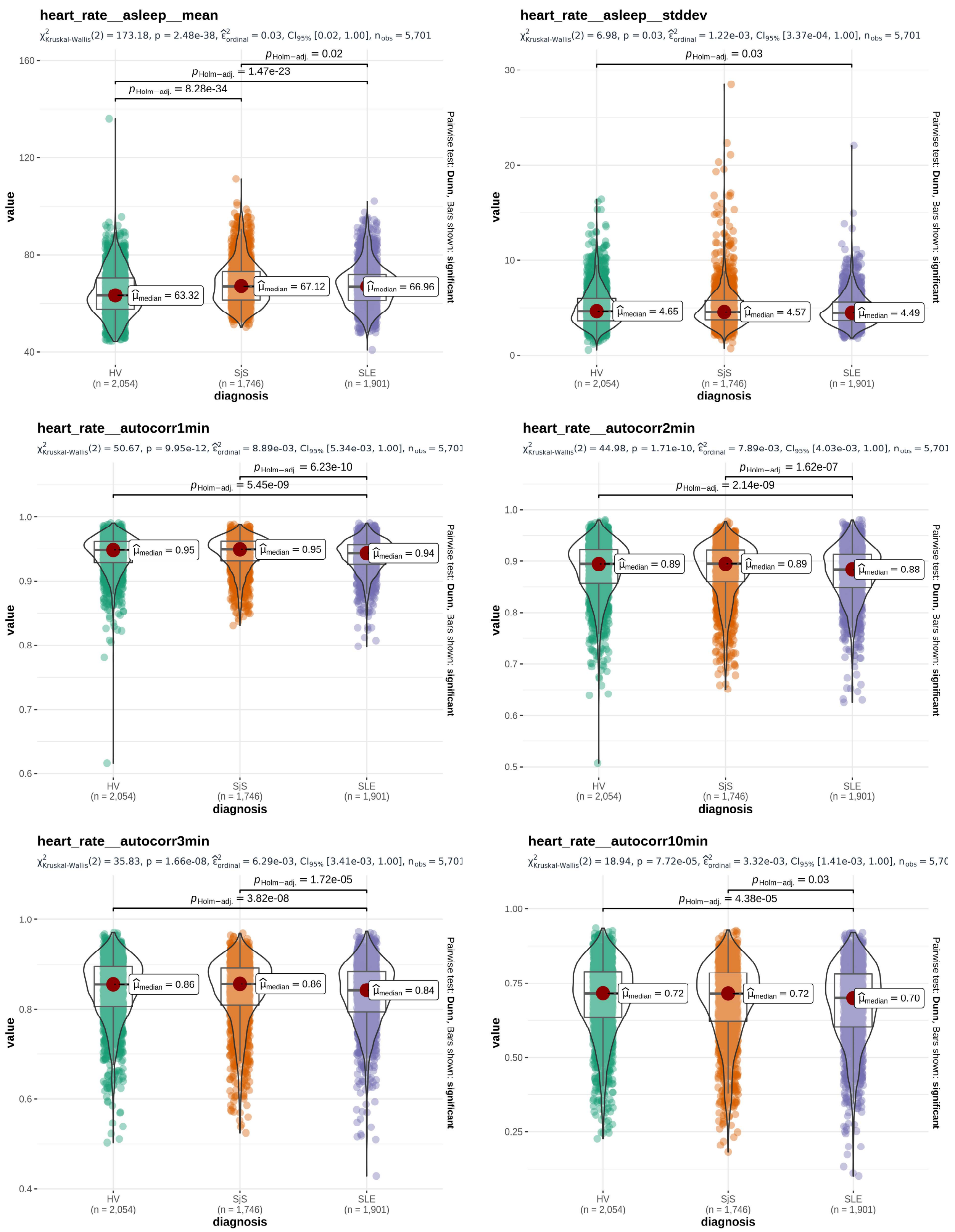

Figure S21: Overlay of violin plots and box plots for all Fitbit features stratified by diagnosis cohorts (HV, SjS and SLE). p-values, adjusted using the Holm-Bonferroni method, from Dunn's pairwise test are reported on the top of each plot and the number of observations (n) at the bottom. Red dots highlight the cohort median values ($\hat{\mu}_{median}$).

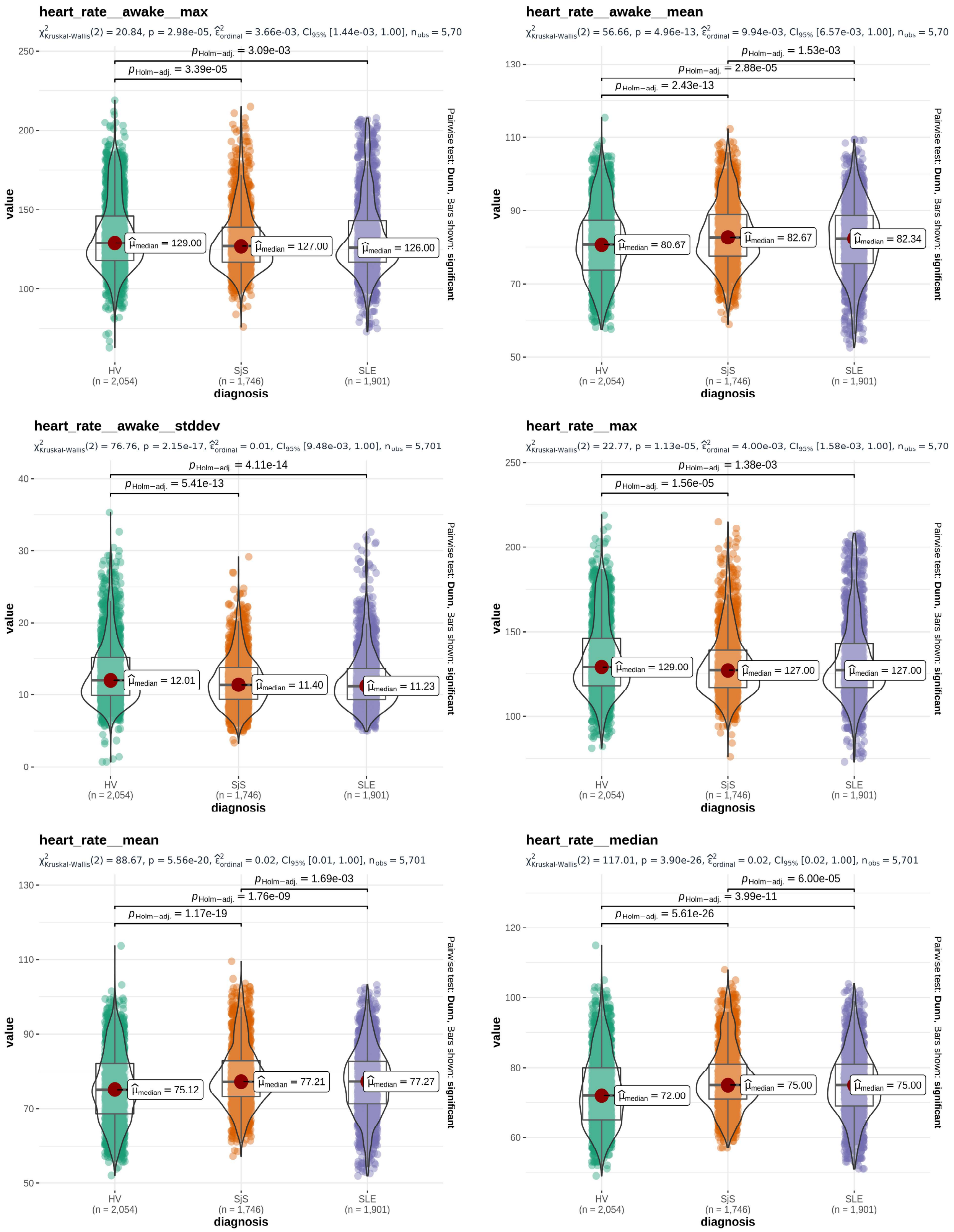

Figure S22: Contd.

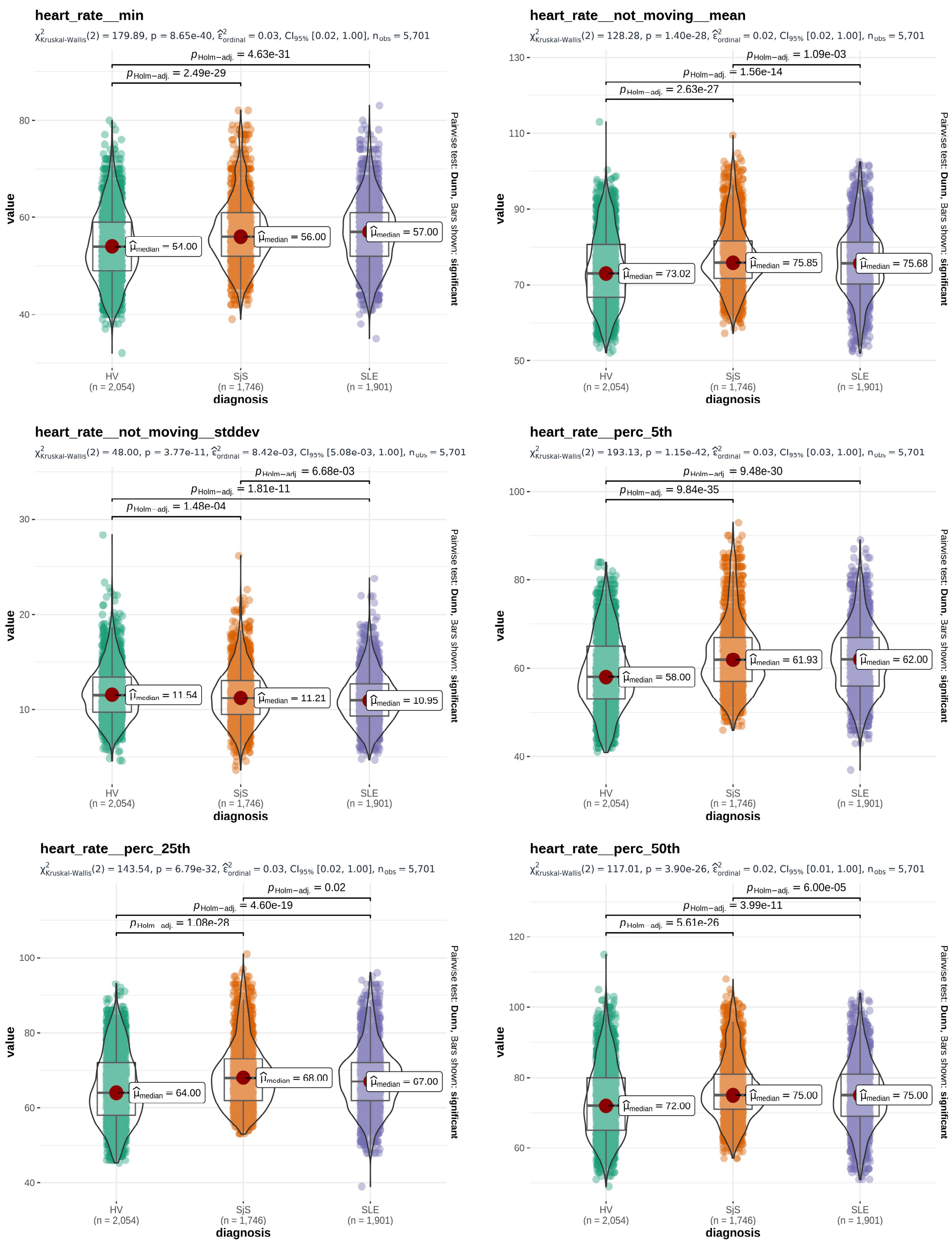

Figure S23: Contd.

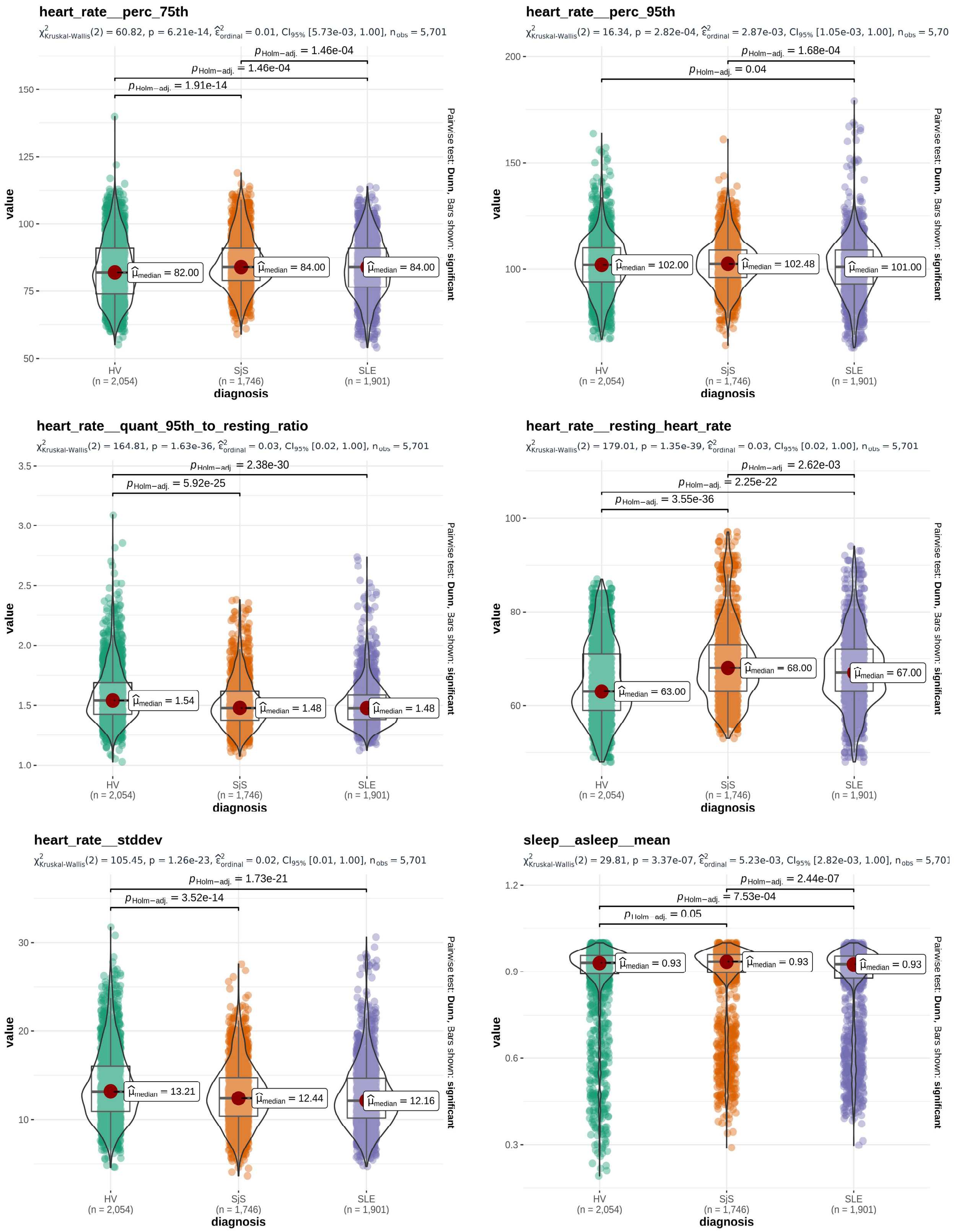

Figure S24: Contd.

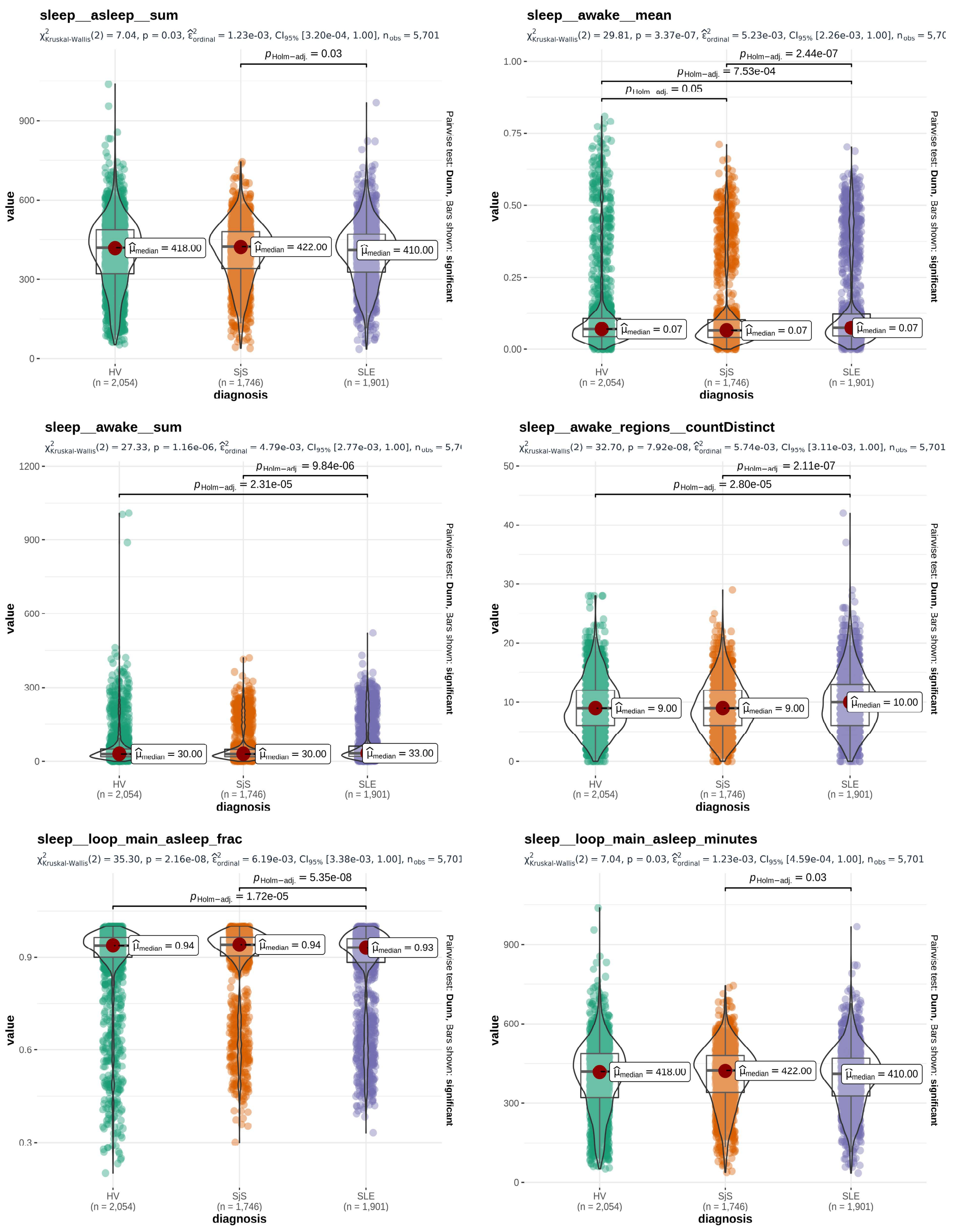

Figure S25: Contd.

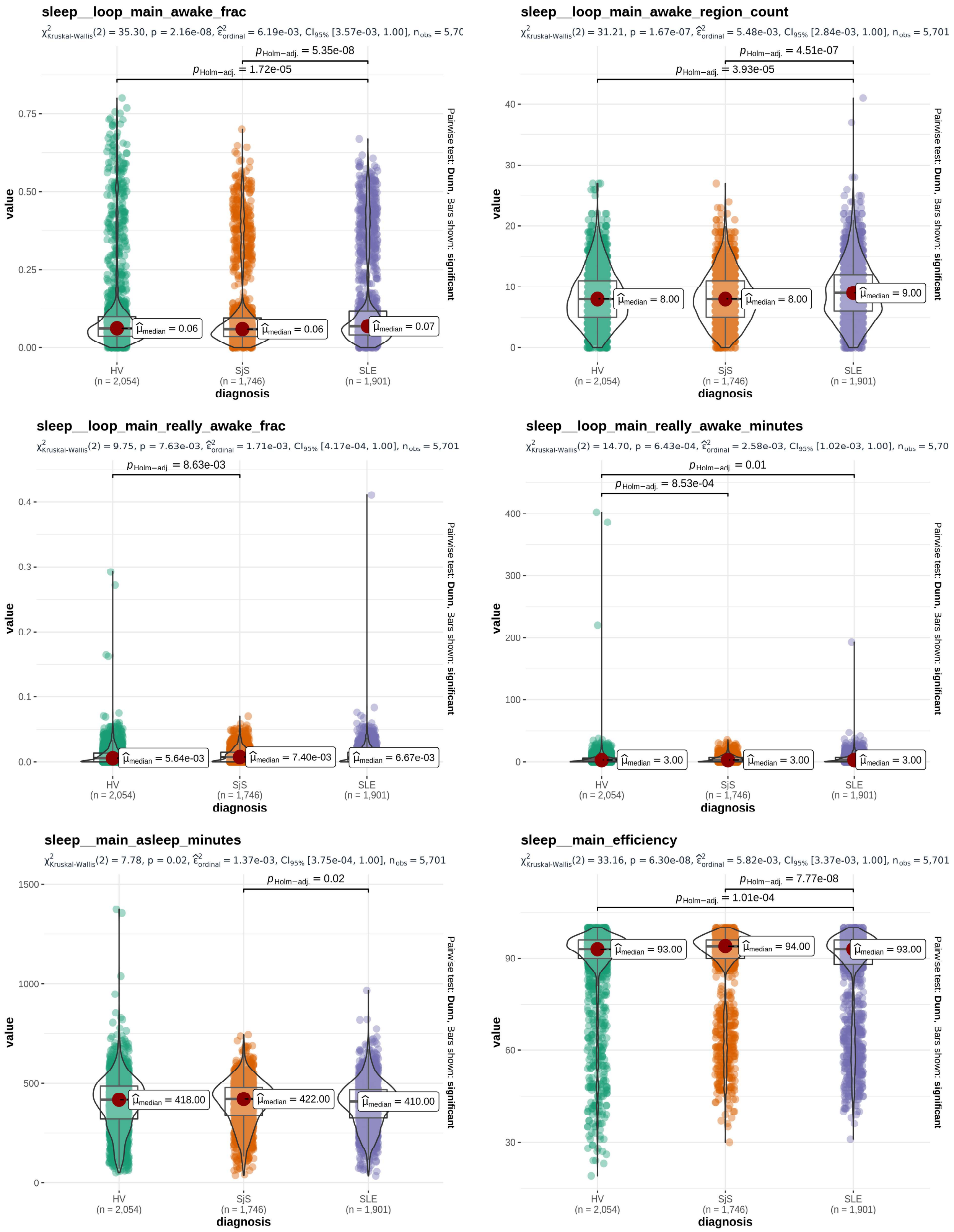

Figure S26: Contd.

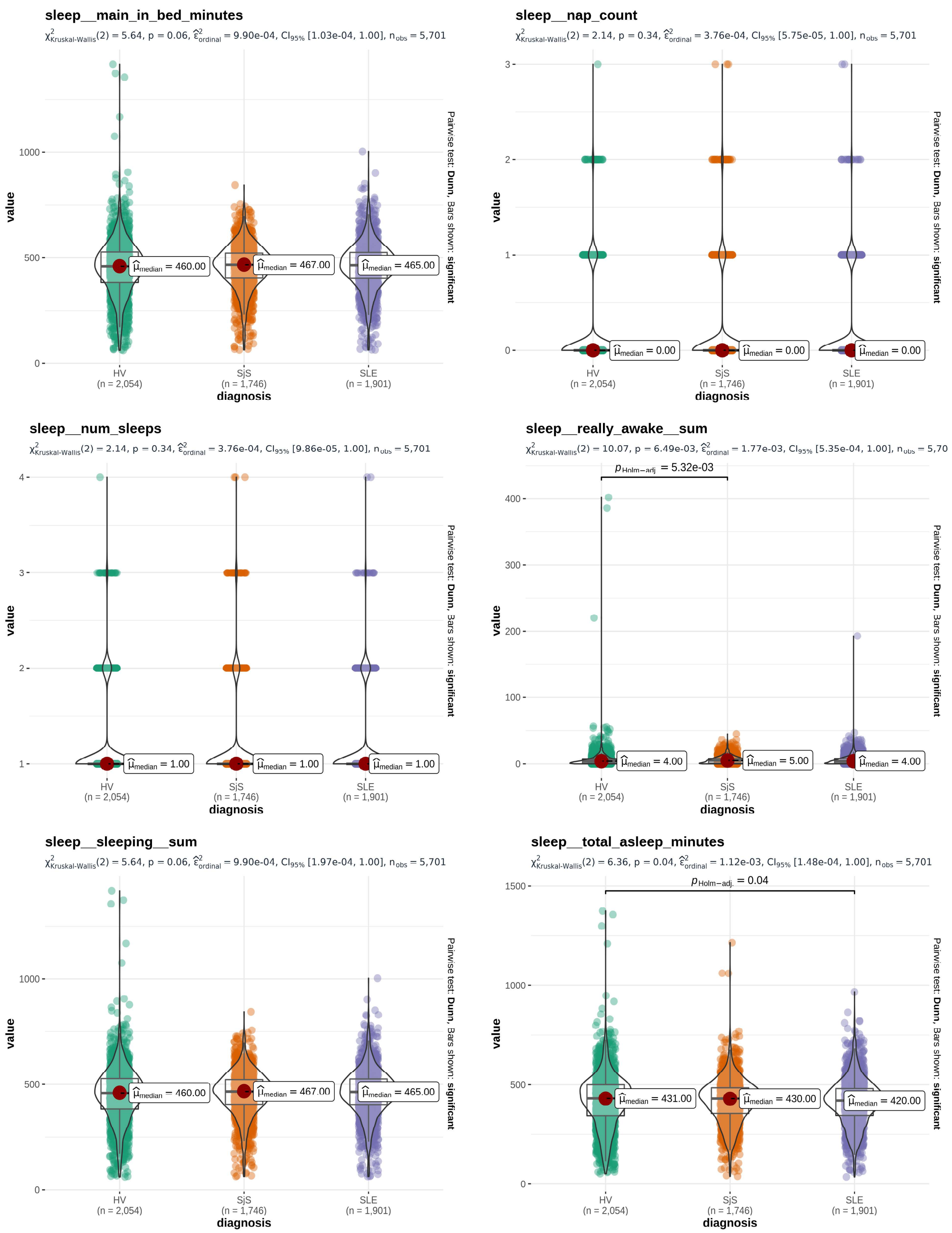

Figure S27: Contd.

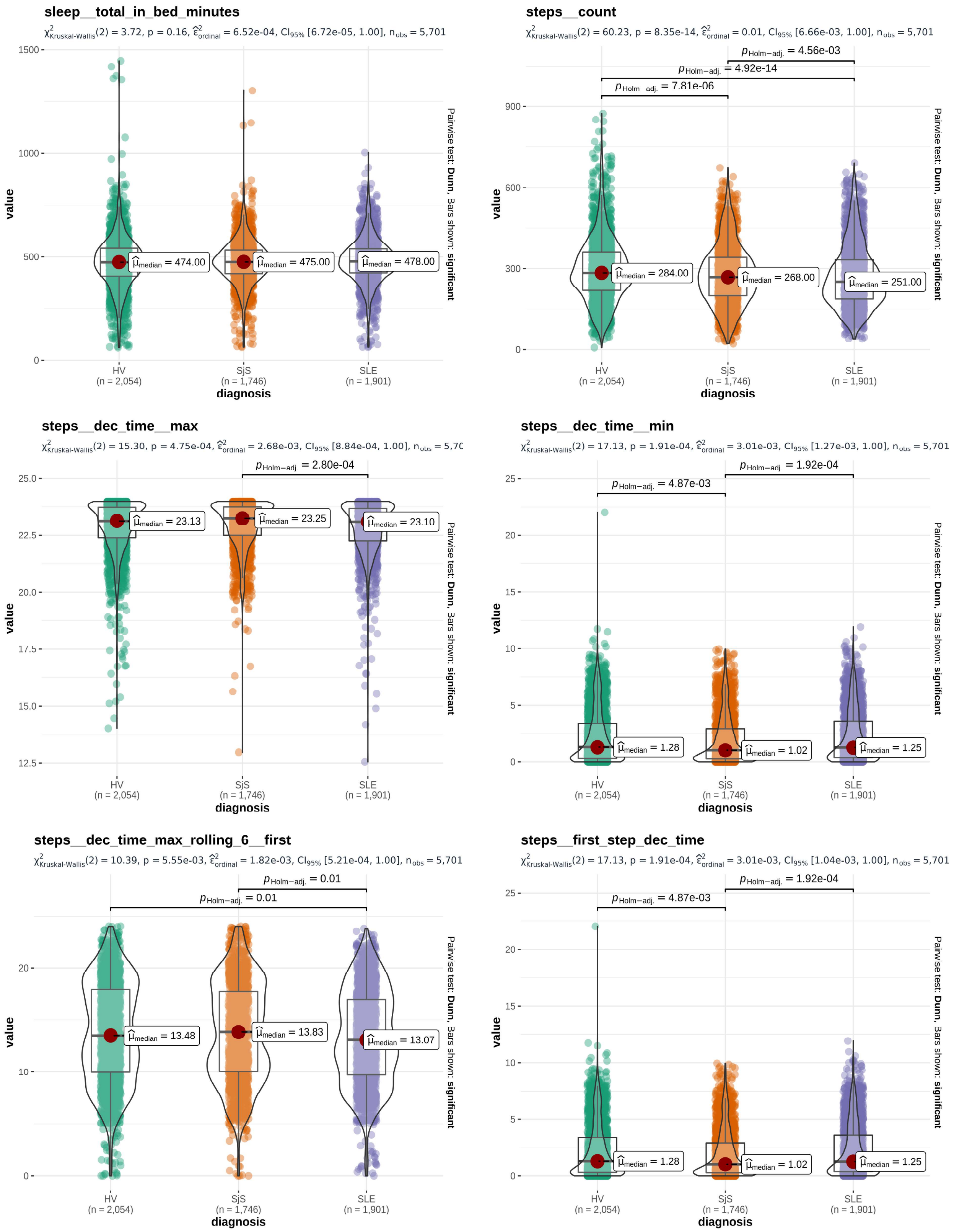

Figure S28: Contd.

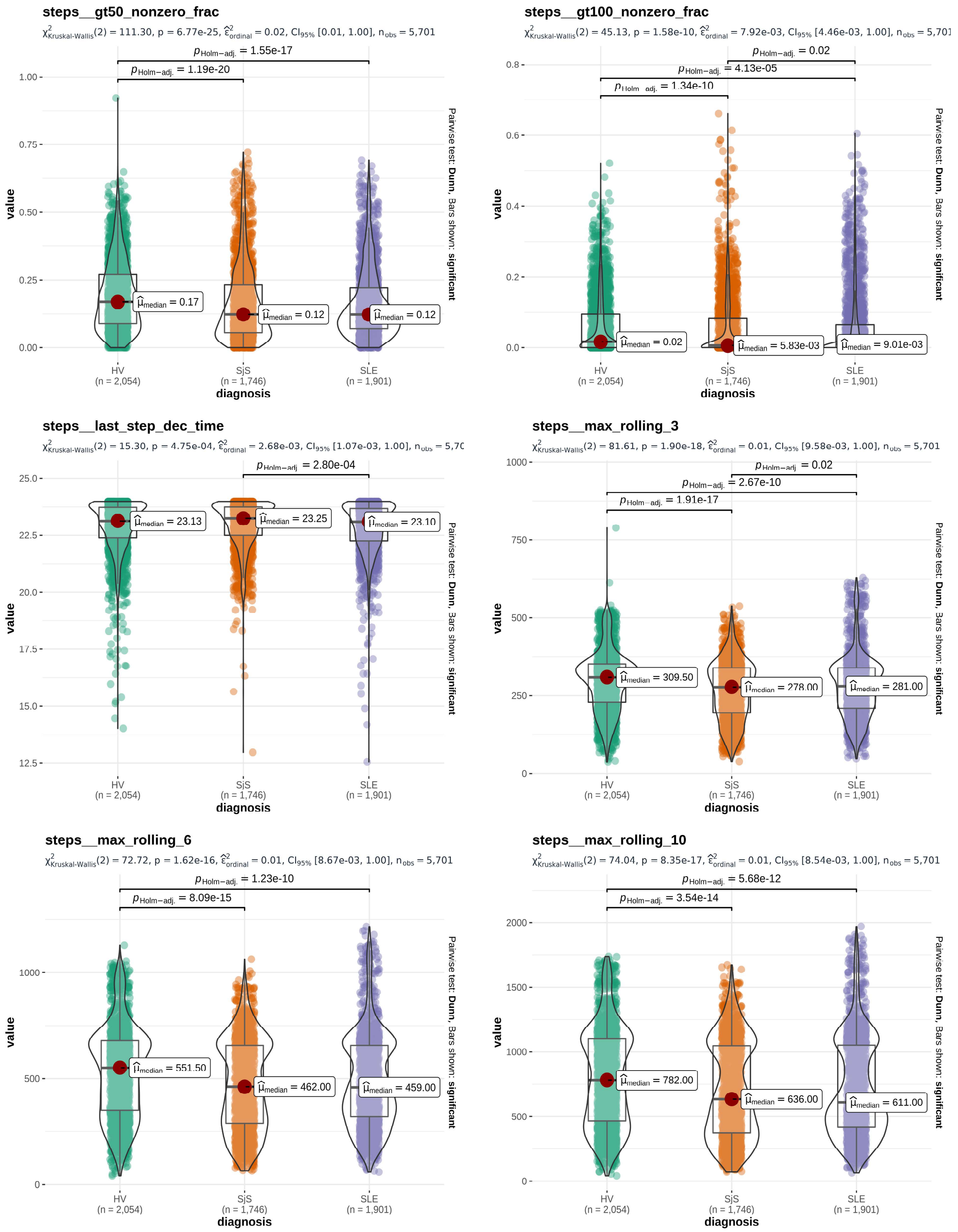

Figure S29: Contd.

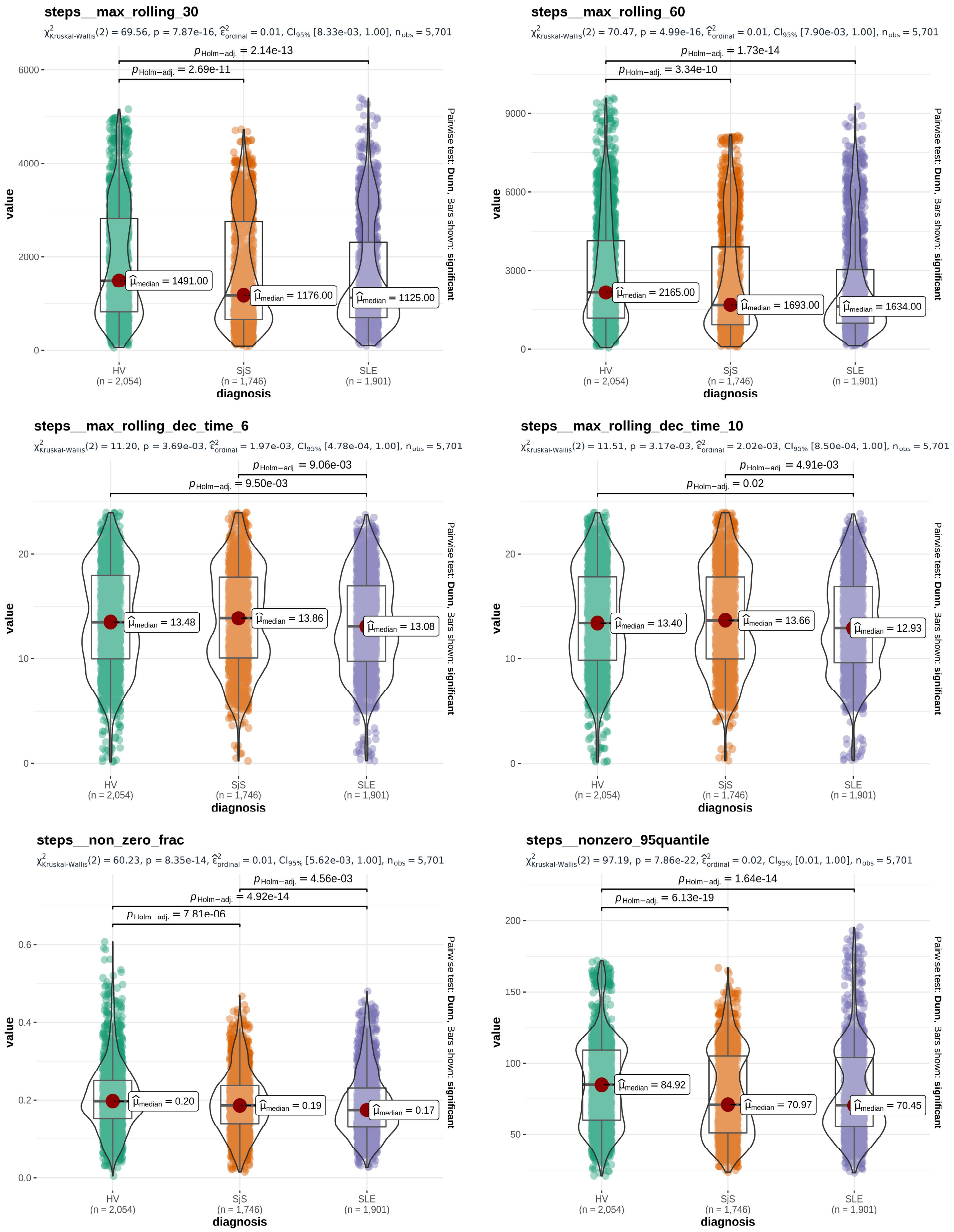

Figure S30: Contd.

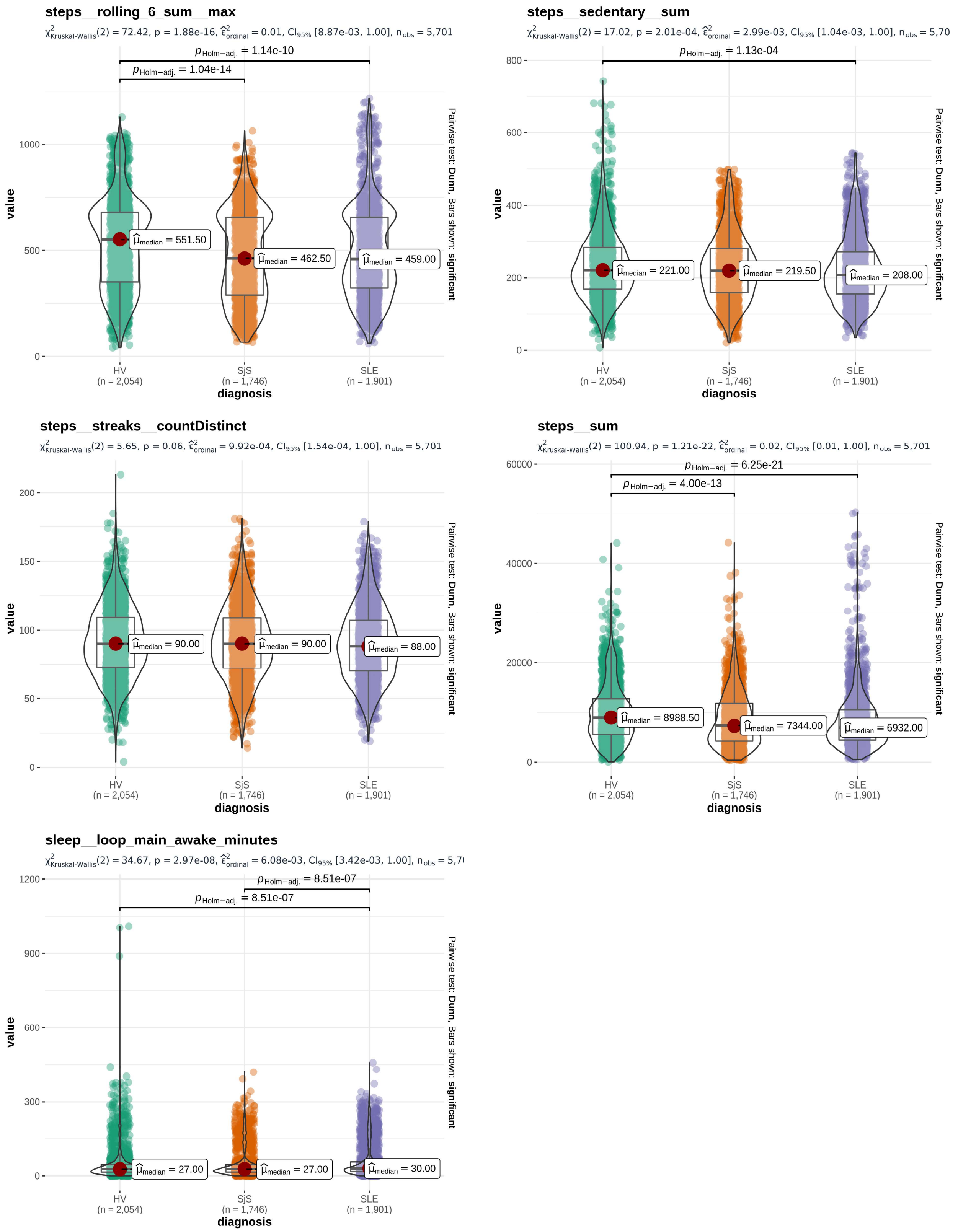

Figure S31: Contd.
