# Supplementary material for: Association of digital measures and self-reported fatigue: a remote observational study in healthy participants and participants with chronic inflammatory rheumatic disease
Source: Front Digit Health. 2023 Jun 22;5:1099456. doi: 10.3389/fdgth.2023.1099456 (PMC10324580; doi:10.3389/fdgth.2023.1099456)
Supplement: Supplementary file 3 [file Datasheet3.pdf]

SUPPLEMENTARY MATERIAL C

7 CLUSTERING RESULTS

In this section, we cover the exploration of the clustering results. Figures S32 and S33 show the hierarchical clustering dendrograms and the cut-off for features and observations, respectively. Table S6 and Figures S35-S38 provides an overview of the characterization of the four clusters with breakdowns on demographics, fatigue labels, ranges of selected Fitibit and SR features.

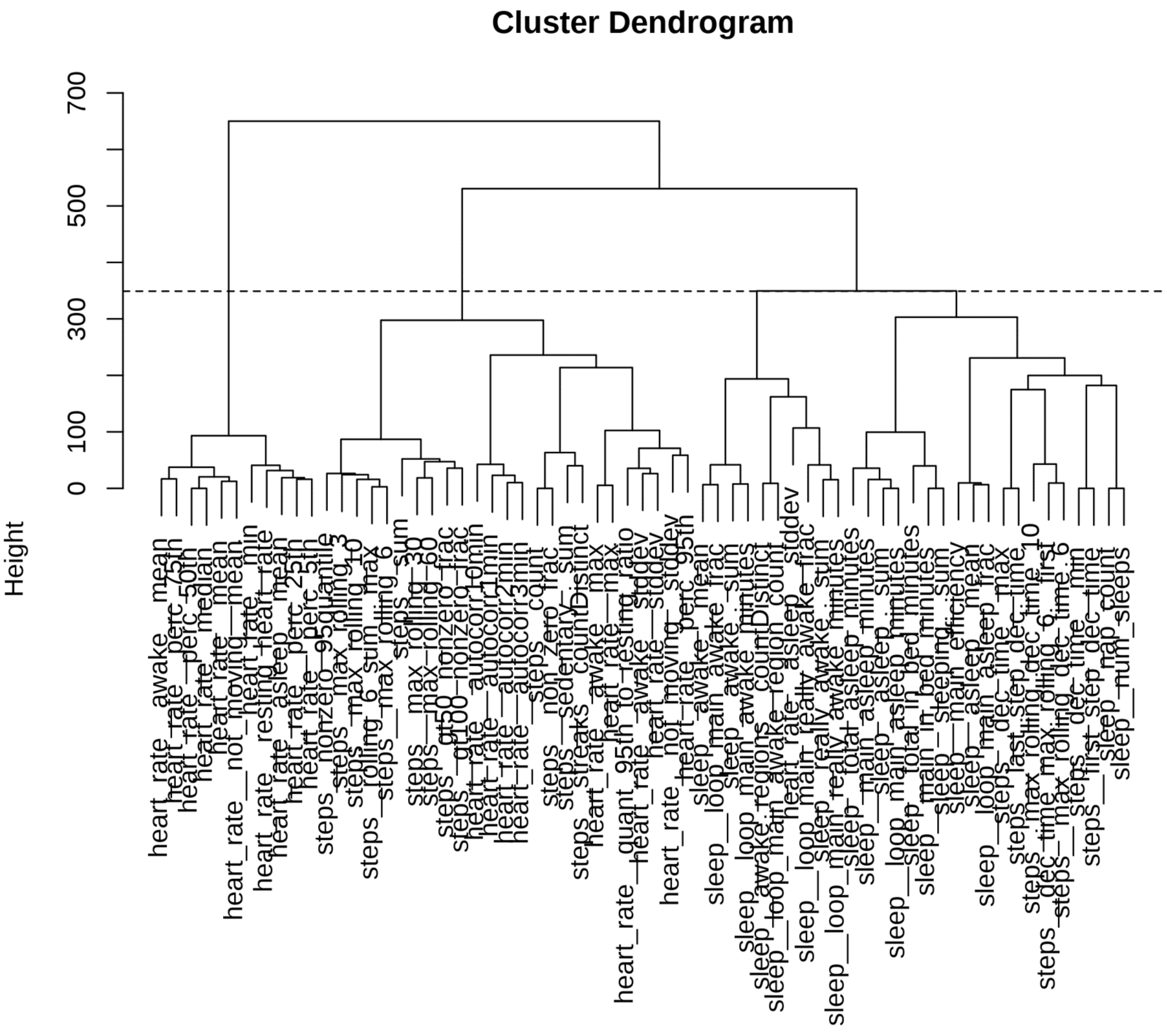

Figure S32: Dendrogram of features, the thresholds used for the heights is 349.

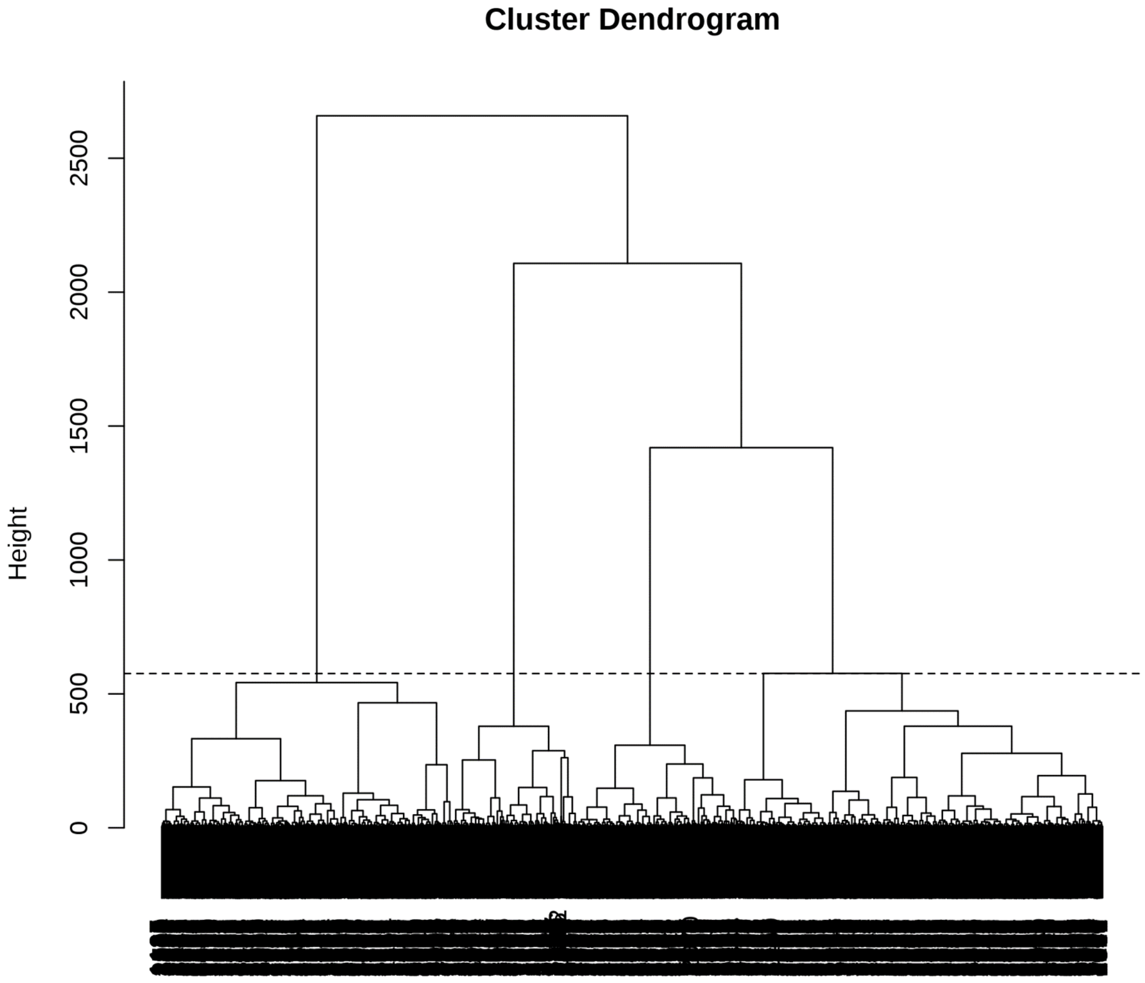

Figure S33: Dendrogram of observations, the thresholds used for the heights is 576.

Table S6. Percentage of observations per participant group in the same cluster.

**Diagnosis**	All observations in same cluster	≥75% of observations in same cluster	≤25% of observations in same cluster
HV	16.18 %	41.18 %	64.71 %
SLE	17.24 %	36.21 %	67.24 %
SjS	28.07 %	43.86 %	57.89 %

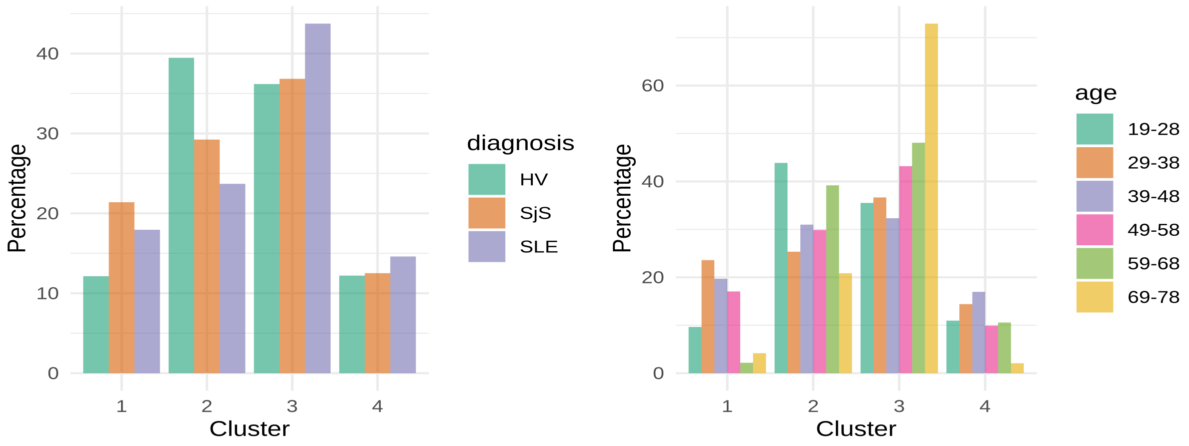

Figure S34: Percentage of participant groups and age range in the 4 clusters.

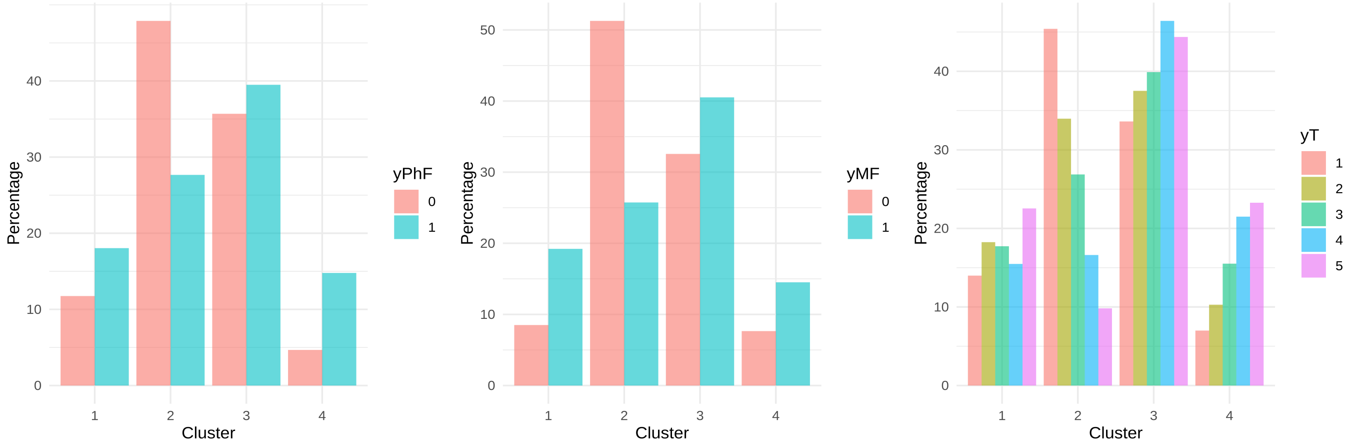

Figure S35: Percentage of binary labels of physical and mental fatigue and ordinal labels of daily tiredness in the 4 clusters.

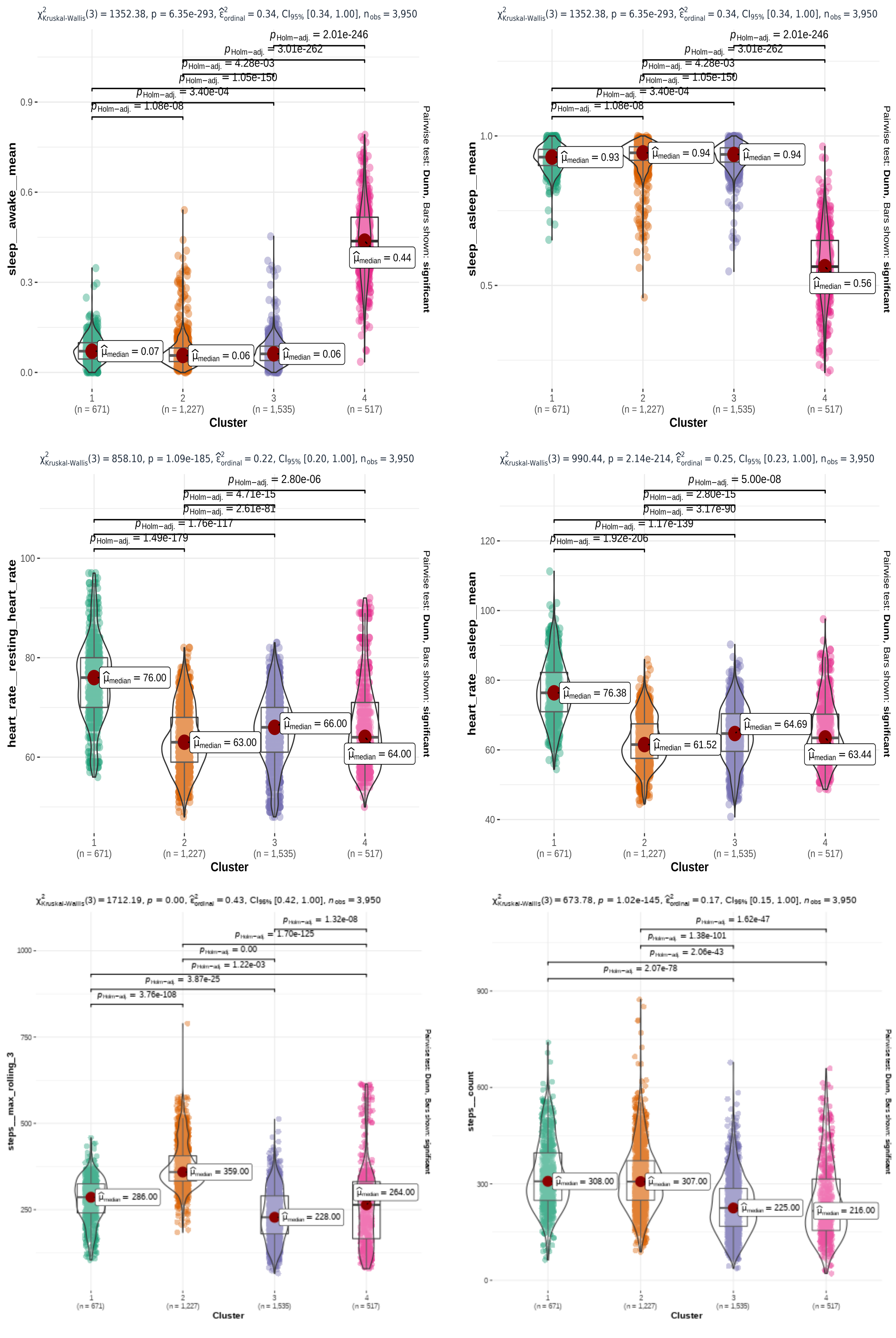

Figure S36: Overlay of violin plots and box plots for some representative Fitbit features that are significantly different among the four clusters. These features were also selected on our previous knowledge of some Fitbit features being significantly different across participants groups. p-values, adjusted using the Holm-Bonferroni method, from Dunn pairwise test are reported on the top of each plot and the number of observations (n) at the bottom. Red dots highlight the cluster median values ($\hat{\mu}_{median}$).

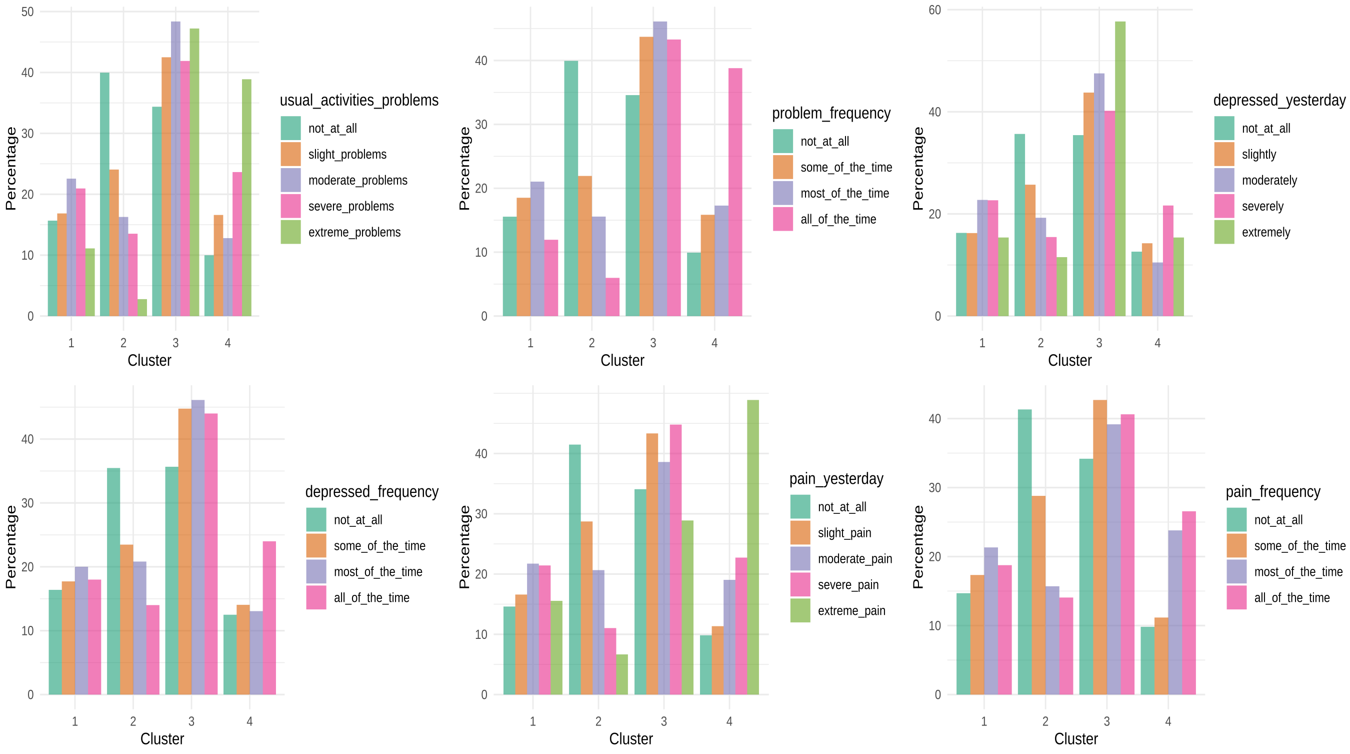

Figure S37: Distribution of participant answers to each PR feature across the four clusters.

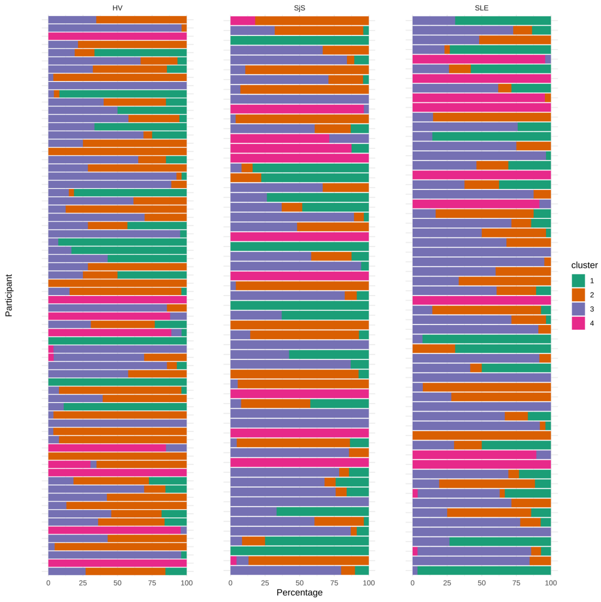

Figure S38: Percentage of observations per participant distributed across the 4 clusters stratified by participant group.

8 IMPLEMENTATION OF CLASSIFIERS

This section reports details of the classifiers' implementation and evaluation.

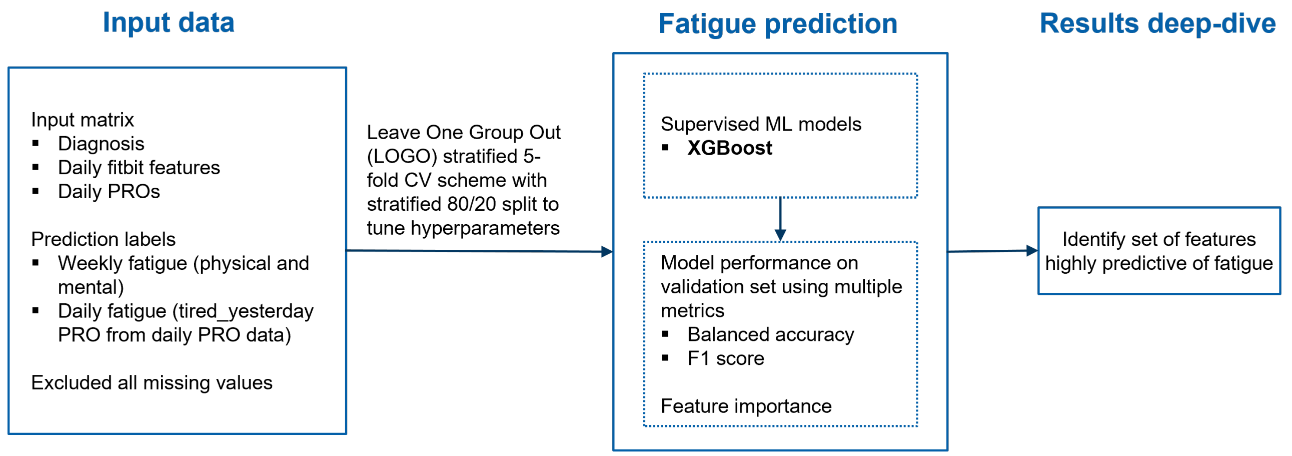

Figure S39: Experimental setup for prediction analysis using XGBoost classifier for binary and multiclass classification.

Library: `https://scikit-learn.org/stable/` v.1.0.2 on Python

Evaluation Metrics: Balanced Accuracy, F1-Score, Macro-averaged Mean Absolute Error

Hyperparameters search of XGBoost:

-Number of trees, "n_estimators" $\in \{100, 200, 300, 400\}$
-Maximum depth of the threes, "max_depth" $\in \{2, 4, 6, 8, 10\}$
-Learning rate, "learning_rate" $\in \{0.05, 0.10, 0.15, 0.20\}$

The library `https://xgboost.readthedocs.io/en/stable/python/python_api.html#xgboost.XGBClassifier` was used for the implementation of XGBoost, hyperparameters not listed in the optimization search were left as default.

Model Training and Evaluation Criteria. A nested 5-fold cross-validation data split was used to evaluate model performance. The outer loop is implemented using a Leave-One-Group-Out (LOGO) strategy ensuring data of the same group of participants not being in the train and test sets concurrently. To select model's hyperparameters a 5-fold cross validation is used within the LOGO train sets (inner loop). The best set of hyperparameters are selected based on the maximum balanced accuracy obtained within each iteration of the inner loop. Considering the unbalanced label class, both inner and outer cross validation followed stratified cross validation, which will keep the split fold with the same percentage of sample for each class. Class weights, in terms of ratio between samples in each class and total number of samples, were added to the loss function of XGBoost during training time to mitigate the class imbalance.

9 CLASSIFICATION RESULTS

This section reports additional details on the classification results, in particular on features importance. Fig. S40 reports the top 20 important features for both mental and physical fatigue classification. Fig. S41 shows the distributions of the top 10 important features for reported level 1 and 5 in daily tiredness.

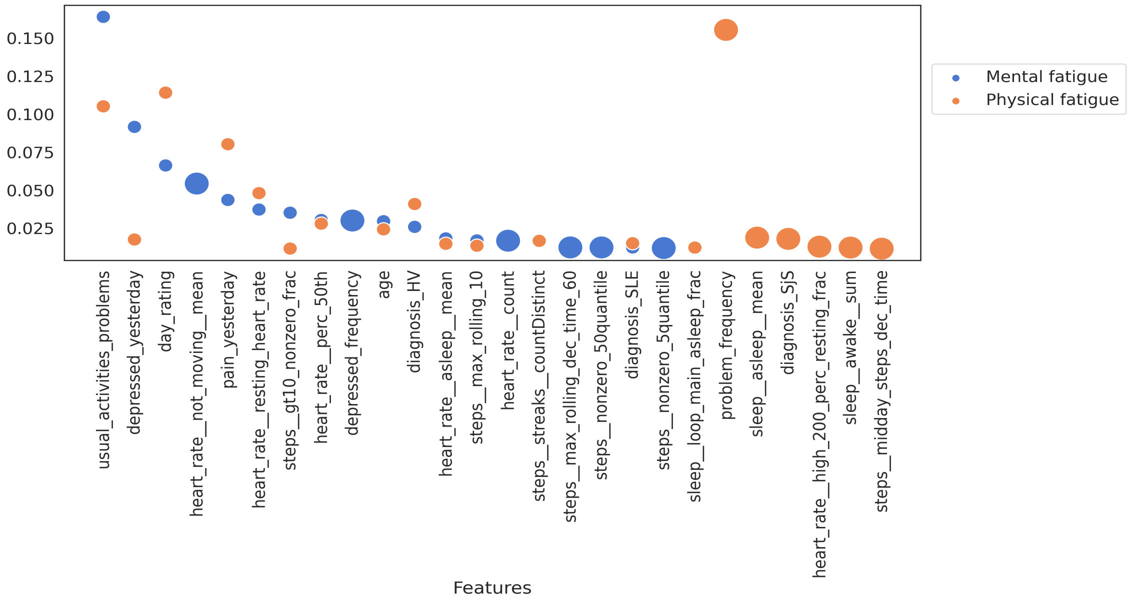

Figure S40: Top 20 important features predictive of binary labels of physical and mental fatigue. y-axis indicates the mean importance across LOGO iterations. The bigger blue points indicate features specific to predicting mental fatigue, the bigger orange points indicate features specific to predicting physical fatigue.

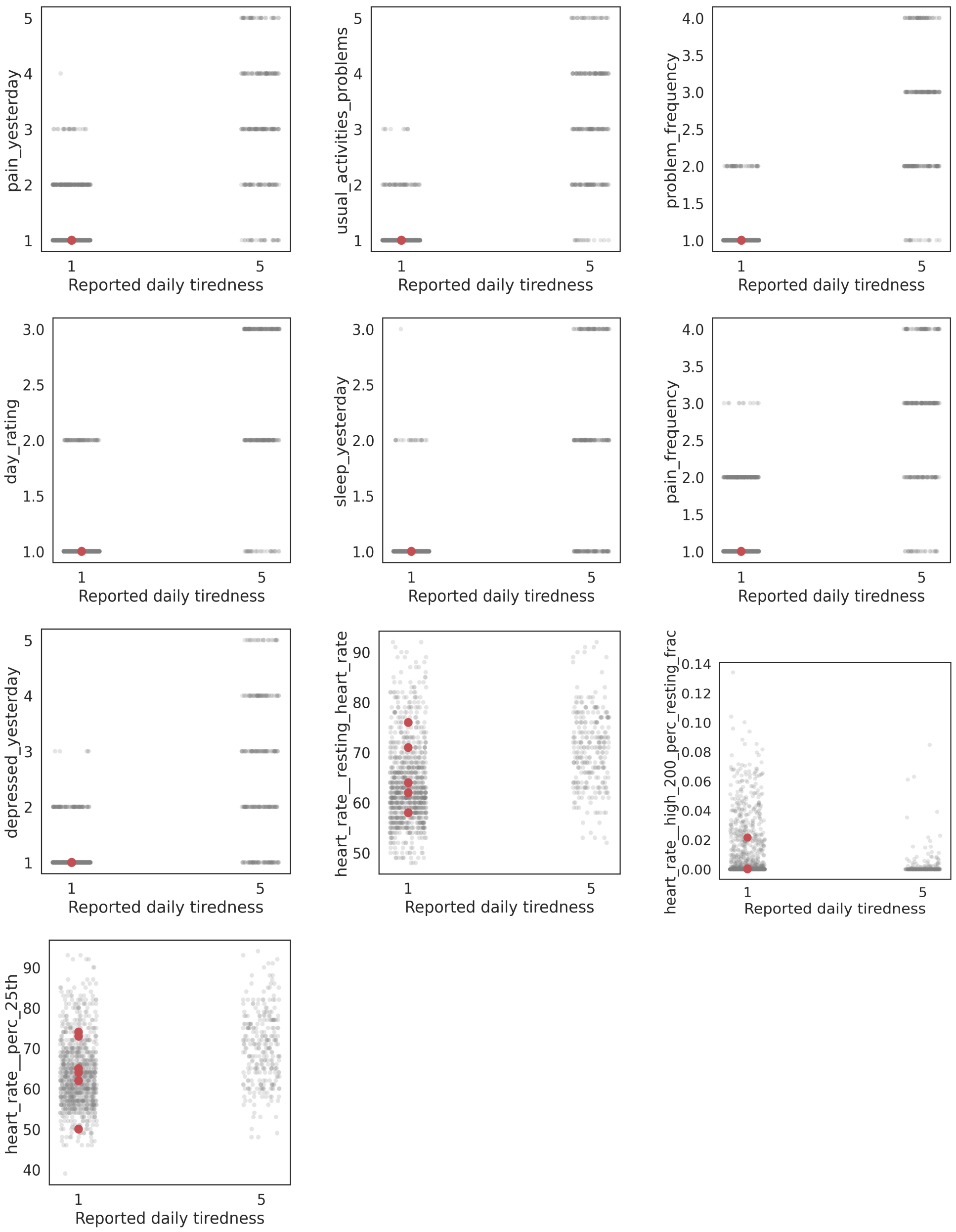

Figure S41: Distribution of top 10 predictive features for levels 1 (corresponding to no fatigue) and 5 (corresponding to the worst fatigue) of reported daily tiredness. The 6 observations marked red in the plot are the misclassified observations, where level 5 was falsely classified as level 1.
